# Impact and Effectiveness of 10 and 13-Valent Pneumococcal Conjugate Vaccines on Hospitalization and Mortality in Children Aged Less than 5 Years in Latin American Countries: A Systematic Review

**DOI:** 10.1371/journal.pone.0166736

**Published:** 2016-12-12

**Authors:** Lucia Helena de Oliveira, Luiz Antonio B. Camacho, Evandro S. F. Coutinho, Martha S. Martinez-Silveira, Ana Flavia Carvalho, Cuauhtemoc Ruiz-Matus, Cristiana M. Toscano

**Affiliations:** 1 Immunization Unit/FGL, Pan American Health Organization, World Health Organization (PAHO), Washington DC, United States of America; 2 Department of Epidemiology and Quantitative Methods in Health, National Public Health School (ENSP), Oswaldo Cruz Foundation (Fiocruz), Rio de Janeiro, Brazil; 3 Library, Gonçalo Moniz Institute, Oswaldo Cruz Foundation (Fiocruz), Salvador, Bahia, Brazil; 4 Vaccine Advocacy and Education, Sabin Vaccine Institute, Washington DC, United States of America; 5 Institute of Tropical Pathology and Public Health (IPTSP), Federal University of Goias (UFG), Goiânia, Goiás, Brazil; Public Health England, UNITED KINGDOM

## Abstract

**Background:**

Several Latin American and Caribbean (LAC) countries have introduced pneumococcal conjugate vaccine (PCV-10 or PCV-13) in their routine national immunization programs.

**Objectives:**

We aimed to summarize the evidence of PCV impact and effectiveness in children under 5 years old in the LAC Region.

**Methods:**

We conducted a systematic review of the literature on impact or effectiveness of PCVs on deaths or hospitalizations due to invasive pneumococcal disease (IPD), pneumonia, meningitis and sepsis. We searched Medline, WoS, Lilacs, Scopus, Central and gray literature published in any language from 2009 to January 2016. We included studies addressing the outcomes of interest in children in the target age group, and with the following designs: randomized trials, cohort or case-control, interrupted time series with at least three data points before and after the intervention, and before-after studies. Screening of citations, data extraction, and risk of bias assessment were conducted in duplicate by independent reviewers, according to the study protocol registered on PROSPERO. Descriptive analysis of the effectiveness measurements and sensitivity analysis were conducted. Effectiveness is reported as 1-OR or 1-RR for case control or cohort/clinical trials, and as percent change of disease incidence rates for before-after studies.

**Results:**

We identified 1,085 citations, 892 from databases and 193 from other sources. Of these, 22 were further analyzed. Studies were from Brazil, Chile, Uruguay, Argentina, Peru and Nicaragua. Effectiveness ranged from 8.8–37.8% for hospitalizations due to X-ray confirmed pneumonia, 7.4–20.6% for clinical pneumonia, and 13.3–87.7% for meningitis hospitalizations, and 56–83.3% for IPD hospitalization, varying by age, outcome definition, type of vaccine and study design.

**Conclusions:**

Available evidence to date indicates significant impact of both PCV-10 and PCV-13 in the outcomes studied, with no evidence of the superiority of one vaccine over the other on pneumonia, IPD or meningitis hospitalization reduction in children under 5 years old.

## Introduction

Pneumococcal diseases are infections caused by *Streptococcus pneumoniae* (*S*. *pneumoniae* or pneumococcus), which is considered the most common vaccine-preventable bacterial etiology of pneumonia, causing approximately 18% of cases in children globally [1]. Worldwide, it was estimated that 14.5 million cases (uncertainty range 11.1–18.0 million) of severe pneumococcal disease occurred each year, resulting in approximately 826,000 deaths (582,000–926,000) [2]. In Latin America and Caribbean (LAC) countries pneumococcus was estimated to cause 12,000–28,000 deaths, 182,000 hospitalizations, and 1.4 million clinic visits annually, in 2009 [3, 4].

The World Health Organization (WHO) in 2012 recommended the introduction of pneumococcal conjugate vaccines (PCV) in childhood immunization programs with high priority to countries with mortality rate >50 deaths/1000 births in children under 5 years of age [5]. The Pan American Health Organization’s (PAHO) Technical Advisory Group (TAG) on vaccine-preventable diseases also recommended in 2011 the introduction of PCV into the Expanded Program on Immunization (EPI) of countries in the American Region [6].

Since 2009 countries in LAC Region have been among the first developing countries to introduce PCVs into their EPIs [7]. As of May 2016, 29 LAC countries and territories were using PCV-10 or PCV-13 with schedules consisting of vaccine doses given at ages 2, 4, and 6 months without a booster dose (3+0), or primary PCV doses administered at ages 2 and 4 months with a booster at age 12–18 months (2+1). Some countries also provided a single catch-up dose to children aged 12–23 months in the year of the vaccine introduction [8].

PCV-10 and PCV-13 were licensed mostly on the basis of comparative immunogenicity with PCV-7, and as such, studies on vaccine efficacy or effectiveness were not available at the time of its initial licensure [5]. Notwithstanding, since the introduction of PCV-10 and PCV-13 in LAC, preliminary evidence suggested that these vaccines were promising in reducing illness and deaths dues to *S*. *pneumonia* [8].

The analysis of variation in the magnitude of the protective effect of PCV vaccines across study settings may be informative of the factors that influence their performance in immunization programs. This systematic review aims at summarizing the evidence of the impact and effectiveness of PCVs on hospitalization and mortality due to pneumonias, meningitis, and invasive pneumococcal disease (IPD) in children less than 5 years old in LAC.

## Methods

The study protocol was registered in PROSPERO under registration number CRD4206032693 (available at http://www.crd.york.ac.uk/PROSPERO/DisplayPDF.php?ID=CRD42016032693). (S1 Appendix)

This study was conducted following the Preferred Reporting Items for Systematic Reviews and Meta-Analyses (PRISMA) statement. (S2 Appendix)

### Literature Search

A systematic literature review was performed to identify all available data from published and unpublished studies conducted in Latin America and Caribbean, on the effects of PCV on hospitalization and mortality in children younger than 5 years of age. Details of the search terms and methods are presented in S3 Appendix. Electronic searches were conducted in the following databases: Medline (PubMed), Scopus, Web of Science, Literatura Latino Americana e do Caribe em Ciências da Saúde (Lilacs), Cochrane Central Register of Controlled Trials (Central), as well as the grey literature, unpublished literature, and selected congress and conference proceedings and annals. There was no restriction regarding languages. Full strategies for grey, unpublished and supplementary searches are presented in S4 Appendix.

### Inclusion and exclusion criteria

We included studies carried out in LAC countries made available (published or presented) between January 2009 and January 2016, without language limitation, with the following study designs: randomized trials, observational studies including cohort and case-control, and quasi-experimental studies including before-after and interrupted time series.

To be eligible for review, studies had to target children aged less than five years of either sex. The study focused on the commercially available pneumococcal conjugate vaccines—PCV-10 and PCV-13—and considered any immunization schedule: 2 primary doses plus a booster (2+1) or 3 primary doses with or without a booster (3+1 or 3+0), with or without catch-up. The outcomes of interest were deaths or hospitalizations due to IPD, pneumonia, meningitis and sepsis. Secondary outcomes, such as serotype specific disease, adverse events, immunogenicity (antibody levels) and nasopharyngeal carriage were considered complementary information. All cause deaths and hospitalizations were not considered as study outcomes.

The following exclusion criteria were considered: cross-sectional studies, case series and case reports as well as studies that only reported data before or after PCV introduction but not for both periods. Interrupted time series studies were included only if they presented data on a minimum of two data points before and after the intervention. For both before-after and time series studies, we excluded those in which only the number of cases were presented, without denominator information or incidence estimates presented. Studies specifically targeted at children with sickle cell disease, HIV-infection or conditions known to affect immune response were not eligible. Studies that considered only disease of selected serotypes, adverse events, immunogenicity (antibody levels), nasopharyngeal carriage, and all-cause mortality and hospitalization as primary outcomes; and studies assessing nosocomial infections were excluded.

### Study Selection

Citations were screened by two independent reviewers in a two-step approach. Titles and abstracts were first reviewed for duplication and inclusion criteria. Duplicates were excluded and full text of those papers meeting inclusion criteria were obtained for completion of screening on their eligibility. Screened articles were categorized as potentially eligible, unclear, or excluded. Citations on which eligibility reviewers disagreed were discussed or assessed by a third reviewer. Reasons for excluding studies were recorded. (S5 Appendix) Authors of studies were contacted when required due to uncertainties or difficulties in decision. Inter-rater agreement (proportion agreement and Kappa statistic) was assessed.

### Data collection and Assessment of Study Quality

Data extraction was done independently by three reviewers, working in pairs, using abstraction forms developed specifically for this systematic review, by study design: cohort, case-control, before-after and time-series.

As recently proposed to assess impact studies, to avoid multiple counting of reports from the same study, citations from the same study group on data originated from the same study protocol, population or information system were grouped for extraction, and reported as a single study [9].

Data extracted included country, funding and ethical issues, study design, intervention details (vaccine used, vaccination schedule, changes in vaccine type), study setting and period, data source, baseline information on study population, case definition and diagnostic criteria, data ascertainment methods, methods for data analysis, and main results including descriptive result and impact assessment results, corresponding confidence limits when available, and any results from sub-group analyses. Additional information on control and ascertainment of exposure for case-control studies and information on loss to follow-up for cohort studies were obtained.

All studies were independently assessed for quality considering the items of structured quality scoring systems as checklists. Data elements of the Newcastle-Ottawa Scale (NOS) [10] were used to address potential sources of bias in case-control and cohort studies, the National Institutes of Health (NIH) checklist for before-after studies [11], and a modified version of Ramsey et al. criteria [12] for time-series studies.

Disagreements between reviewers were assessed and the major sources of divergence discussed until agreement was reached. When disagreement was not resolved, a third reviewer was used as an arbitrator.

### Data analysis

This paper presents data on effects of PCV on a variety of outcomes. As such, study results are presented by the following outcomes: pneumonia, meningitis, and invasive pneumococcal disease. As pneumonia studies included different case definitions, results were further grouped by the following categories: X-ray confirmed pneumonia (consolidated), and clinical pneumonia (including broad pneumonia definitions based on ICD10 codes J12-J18 when using secondary data sources, and cough and fever as case definition when primary data from surveillance was used).

A descriptive analysis of study characteristics including design, country, type and schedule of PCV introduced, data source, and endpoints considered was conducted. All results were stratified by age groups as presented by authors.

For all studies, the main measure of interest was the vaccine effect in reducing the outcome of interest. In case control and cohort studies, this was reported as odds-ratio (OR) and relative risk (RR), and the resulting effectiveness was estimated as 1-OR or 1-RR. For time series studies, the effect was reported as either the % reduction in rates when modeling observed rates against predicted rates of disease, or resulting from a percent change in incidence rates when comparing the post and pre-vaccination periods.

In before-after studies, vaccine effects were reported as % change in rates (incidence or mortality rate reduction). Studies reporting only number of cases were excluded from the analysis as it was not possible to determine the impact of the vaccine as a rate reduction. When possible, we calculated the percent change in rates using a systematic method in which all data points in the pre and post intervention period were considered, and the intervention year was excluded from the analysis. Percent change was calculated as ((pre PCV incidence rate–post PCV incidence rate)/pre PCV incidence) * 100. We conducted all analysis using Stata 10.0 (StataCorp. 2007. *Stata Statistical Software*: *Release 10*. College Station, TX: StataCorp LP.).

The level of risk of bias in study analysis was assessed for each study expanding on the structured assessment using published scales and checklists [11–13]. Internal validity of each study was evaluated considering six methodological domains [14]: selection of study participants, exposure and outcome variable measurement, design-specific sources of bias, control of confounding, statistical analyses, and conflict of interest.

Results are reported as intervals of VE by endpoint (pneumonia, meningitis, IPD) and age group. Estimates of VE on hospitalization and mortality are presented separately. A posteriori, we defined the following age groups for which results are reported: <12m, 12-23m, 2-23m, 12-35m, 0-35m, 24-35m, 24-47m, 24-59m, <48m, and <60m. Further, combined results including all age groups < 2 years, the most important target age group for PCV, are presented.

Sensitivity analysis was conducted to assess the impact of excluding studies which included pneumonia inpatients and outpatients combined, and selected outlier estimates from studies with significant potential for bias.

## Results

**Fig 1:**
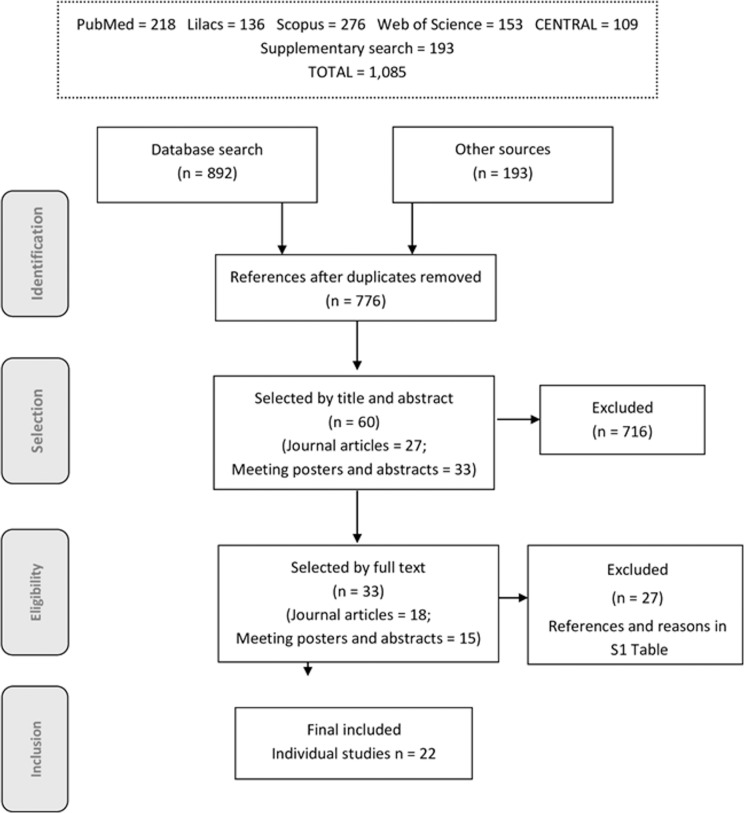
Flowchart: process of study selection.

A total of 1,085 references were identified and 33 were eligible for data extraction. They comprised 18 full reports and 15 abstracts and posters, reporting on 22 individual studies (Fig 1). Of the 33 references, 11 were multiple reports of the same studies. An additional 2 pairs of studies [15, 16] reported on the same population and surveillance data, but used different methods for data analysis. In one case, two studies by the same authors [17, 18] reported on cases from the same area/health services in overlapping periods, but differed in approach (individual vs. aggregate data) and reference group (unvaccinated group with concurrent follow-up data vs. surveillance data from population long before PCV was available). Also related in their dataset were the matched case-control study by Domingues et al. [15] and the ancillary study by Verani et al. [16], based on cases only (vaccine-type and vaccine related cases) compared to non-vaccine-type disease (“indirect cohort method”).

Of the 22 studies that met the inclusion criteria 5 were only available as poster/abstract. Studies were conducted in Argentina, Brazil, Chile, Nicaragua, Peru and Uruguay, with more than half of them being from Brazil. No study from the Caribbean countries was included. Most studies were published/presented in 2014–2016 (91%). PCV-10 was assessed in most of the studies (68%), and the predominant dosing schedules, regardless of vaccine type, were 3+1 and 2+1 (95%). A variety of endpoints, study designs and data sources were considered. Pneumonia was the most frequent endpoint of interest, and no study evaluating sepsis as an individual outcome was included in this review. Before-after was the most common study design, and surveillance the most common data source used in the reported studies (Table 1). A total of 6 studies analyzed mortality: 2 evaluated PCV impact on pneumococcal meningitis deaths [19, 20], 2 evaluated impact on all-cause deaths [21, 22], and 3 analyzed deaths due to pneumonia [22–24].

**Table 1:** Summary of characteristics from 22 included studies.

Characteristics	n (Total 22)	%
Publication Type		
Full report	17	77
Abstract	5	23
Publication Year		
2012	1	5
2013	1	5
2014	9	41
2015	7	32
2016	4	18
PCV product		
PCV-10	15	68
PCV-13	7	32
Dosing schedules		
2+1	9	41
3+0	1	5
3+1	12	55
Country		
Argentina	3	14
Brazil	12	55
Chile	2	9
Nicaragua	1	5
Peru	1	5
Uruguay	3	14
Endpoint*		
Pneumonia	12	55
X ray confirmed	5	23
Clinically confirmed	6	27
Both	1	5
IPD	5	23
Meningitis	5	23
Study Type		
Before-after	11	50
Time series analysis	7	32
Case control	2	9
Cohort	1	5
Indirect cohort^&^	1	5
Data sources		
Surveillance	16	73
Secondary hospitalization data	6	27

* One study may consider more than one endpoint

^&^ Also denominated case-only study by some authors

The variety of study designs and methods used in the studies made it inappropriate to conduct a meta-analysis.

### PCV effectiveness on pneumonia hospitalizations and deaths

Thirteen of the studies included in this review evaluated PCV effectiveness on pneumonia (Table 2). Among those studies, 8 evaluated pneumonia hospitalizations only [17, 18, 21, 22, 25–28], 4 evaluated both hospitalized and outpatient pneumonia combined [24, 29–31] two evaluated both pneumonia hospitalizations and deaths [22, 24], and one evaluated only pneumonia deaths [23]. Among the 13 studies, there were 5 interrupted times series [21, 23–25, 32], 6 before-after [17, 27–30], 1 cohort [18] and 1 case-control study [22].

**Table 2:** Characteristics of studies reviewed with pneumococcal pneumonia as endpoint.

First Author, year	Country^$^	Vaccine	Study design	Case definition	Data source	Age * groups	Years of baseline data	Baseline measure (Rates p. 100,000)	Years of post PCV introduction data	Percent change/effectiveness	Statistical Significance (95% CI or p-value)
Afonso, 2013 [25]	Brazil (5 capitals)	PCV-10	Interrupted time series	ICD10 codes J12-18	Secondary Hospitalization Data	2-24m	Jan05-Aug11	Belo Horizonte 164.3	1	40	27.4–50.9
								Curitiba 79.0		37.6	22.7–49.6
								Recife 130.4		49.3	33.1-61-6
								São Paulo 124.7		13.4	-1.42–26.02
								Porto Alegre 29.1		23.5	-0.2–41.6
Suarez, 2016 [24]	Peru	PCV-10	Interrupted time series	ICD10 codes J12-18 –(inpatients and outpatients)	Secondary Data	<12m	Jan06-Dec08	58.0	2	20.6	10.9–29.5
				Death due to pneumonia (ICD10 codes J12-18)				8	2	35.0	8.6–53.8
Becker-Dreps, 2013 [33], 2014[21]	Nicaragua (León Department)	PCV-13	Interrupted time series	X-ray confirmed pneumonia	Hospital population based surveillance	<12m	Jan08-Dec10	6440^&^	2	33	25–41
						12-23m		2490^&^		26	19–33
						24-59m				27	19–34
Diaz, 2016 [22]	Chile	PCV-10	Nested case control	ICD10 codes J13-18	Secondary Hospitalization Data	2-23m	2010/2012^#a^	13,210 cases and 52,840 controls	2^#b^	20.7	17.3–23.8
				Death due to pneumonia (ICD10 codes J13-18)	Secondary Hospitalization Data	2-23m	2010–11^#a^	36 cases and 144 controls	2^#b^	71.5	9–91.8
Hortal, 2014 [17]	Uruguay (2 municipalities)	PCV-13	Before-after	X-ray confirmed pneumonia	Hospital population based surveillance	<12m	2001–2004	2604^&^	4	8.8	NS
						12-23m		2383^&^		37.8	<0.001
						24-35m		1349^&^		24	<0.05
						<60m		1542^&^		20.4	<0.001
Hortal, 2014 [34], 2015 [18] ^&^	Uruguay (2 municipalities)	PCV-13	Cohort	X-ray confirmed pneumonia	Hospital population based surveillance	0-35m	2010^#a^	1048^&^ vaccinated; 5679^&^ non vaccinated	3^#b^	84.6	NR
Sgambatti, 2014 [28], 2016 [35]	Brazil (Goiania municipality)	PCV-10	Before-after	X-ray confirmed pneumonia	Hospital based surveillance	<12m	2007–2009	678.8	3	25.3	24.6–26.1
						12-23m		480.2		25.1	24–26
						24-35m		240.8		11.9	11.3–12.7
				Clinical pneumonia							
						<12m		287.1		12.6	12.3–12.9
						12-23m		215.1		14.2	13.7–14.6
						24-35m		100.9		7.4	7.1–7.8
Scotta, 2014 [27], Pinto, 2013, [36]	Brazil	PCV-10	Before-after	ICD10 codes J12-J18	Secondary Hospitalization Data	<12m	2002–2009		2	10.4	NR
						12-35m				14.2	NR
						<48m		2800^&^		12.7	NR
Gentile, 2014 [38], 2015 [30], 2015 [37]	Argentina (Pilar municipality)	PCV-13	Before-after	X-ray confirmed pneumonia (inpatients and outpatients)	Hospital population based surveillance	<12m	2003–2005	1922^&^	1	44.6	24.6–59.3
						12-23m		931^&^		57.9	31.1–74.2
						24-59m		321^&^		18.8	NS
						<60m		750^&^		39.6	25.0–51.3
Gaiano; 2013 [29], Vizzotti, 2014 [39], 2014 [40]	Argentina	PCV-13	Before-after	Clinical pneumonia—(inpatients and outpatients)	Secondary data	<12m	2011	2880	1	28.06	26.5–29.6
						12-24m		2370		30.34	28.9–31.8
Rearte, 2015 [31] ABSTRACT ONLY	Argentina (Concordia municipality)	PCV-13	Before-after	X-day confirmed pneumonia (inpatients and outpatients)	Hospital population based surveillance	<60m	2002–2005	732	1	50.7	33–64
Andrade, 2015 [26] POSTER ONLY	Brazil	PCV-10	Interrupted time series	ICD-10 codes J12-J18	Secondary Hospitalization Data	2-23m	2005–2009	Not shown	3	16.6	1.0–32.1 -
						2-11m				13.6 20	3.0–24.3 2.3
						12-23m				20.2	381 0.7
						24-60m				14.4	28.1
Minamisava et al., 2014 [23] ABSTRACT ONLY	Brazil	PCV-10	Interrupted time series	Death due to pneumonia (ICD10 codes J12-J18)	Mortality Information System	2-23m	2005–2009	Not shown	2	15.5	-7.2–38.2

* Age groups with results of interest for this study; ^#^ VT-PCV-13; ^&^ rates per person-years

^**$**^: When not specified otherwise, country of study refers to nationwide data.

#a: Start of case detection

#b: duration of follow-up/case detection

^**&**^: This study reports on the same data/study population as the above (Hortal, 2014) [17] using different study method and considering 2010–2013 as opposed to 2009–2012 post–vaccine period data in the analysis

Hospitalization rates for pneumonia in 13 studies varied widely (29.2 to 2880 per 100,000 and 321 to 6,440 per 100,000 person-years), mainly by outcome (inpatients only vs. inpatients and outpatients combined), and age subgroups (Table 2).

**Fig 2:**
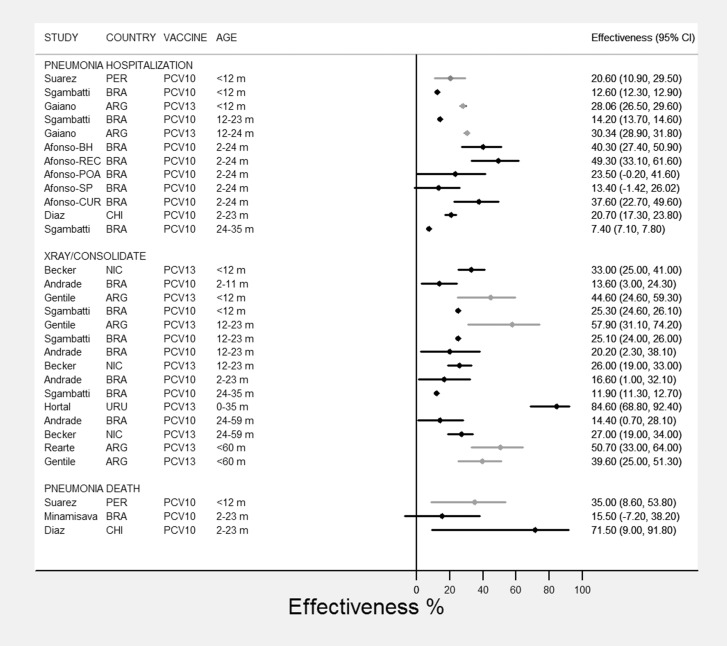
Vaccine effectiveness (%, 95% confidence interval*) against pneumonias clinical and X-Ray/consolidated, by vaccine, group of age, and hospitalization or death. * Effectiveness estimates and 95%CI are presented in black for studies assessing hospitalized pneumonia, and in light gray for studies assessing combined pneumonia inpatient and outpatients as endpoints. Two studies with no available confidence intervals were not plotted: Hortal et al.[17]; Scotta et al.[27] Countries: ARG (Argentina); BRA (Brazil); CHI (Chile); NIC (Nicaragua); PER (Peru); URU (Uruguay).

All included studies on pneumonia hospitalization and deaths reported 35 effectiveness estimates in different age subgroups. When we consider all the above reports on PCV-10 and PCV-13 effectiveness (VE), regardless of outcome, age group, data source and study methodology, VE point estimates varied from a lower 7.4% to 84.6%. Some of the estimates had very wide confidence intervals (Fig 2), whereas two studies [18, 27] presented only point estimates. Another study did not present confidence intervals but reported statistical significance (p-values or “NS”) [17]. Of note, VE on X-ray confirmed hospitalized pneumonias reported by Hortal et al. [17] using before-after analysis varied from 8.8 (non- significant) in the <12-month age group to 37.8% (p-value < 0.001) in the 12–23 month group. However, estimates using a cohort study design analyzing data obtained by the same surveillance system in the same study population found VE estimates of 84.6% (95% CI not reported) for the <3 year age group. [18]

When results are stratified by the different pneumonia case definitions, regardless of age subgroup, PCV-10 and PCV-13 effectiveness estimates for clinical pneumonia varied from 7.4% to 49.3%, whereas for consolidated (X-ray confirmed) pneumonia VE ranged from 8.8% to 84.6%.

In sensitivity analysis, disregarding the outlier estimate reported by Hortal [18], VE for X-ray confirmed pneumonia ranged from 8.8% to 57.9%. In further sensitivity analysis, including only studies assessing hospitalized pneumonia (effectiveness estimates and CI presented in black in Fig 2), and disregarding estimates for combined pneumonia inpatient and outpatient endpoints [24, 29–31] (presented in light gray in Fig 2), VE ranged from 7.4% to 49.3%. Finally, VE estimates reported by Afonso et al. [25] with a very short follow-up period after PCV introduction were also identified as outliers prone to biases. When these results were excluded, ranges of VE were 7.4%-20.6% for clinical pneumonia, and 8.8%-37.8% for X-ray confirmed (consolidated) pneumonia.

When only effectiveness estimates from the studies included in sensitivity analyses were stratified by vaccine type, for clinical pneumonia outcome, VE varied from 7.4 to 20.6% among the various age subgroups. Those results concern studies conducted in sites using PCV-10 (n = 4) as studies from sites using PCV-13 did not fulfill the criteria above for sensitivity analysis (i.e., assessing only pneumonia hospitalizations). When X-ray confirmed pneumonia (consolidated) outcome was considered, estimates varied from 11.9% to 25.3% for PCV-10 (n = 6), and from 8.8 to 37.8% for PCV-13 (n = 2). Estimates for both PCV vaccines in either pneumonia outcome fell under overlapping 95% confidence limits (Fig 2).

Given the great diversity of age subgroups in the reports (Table 2; Fig 2), stratification resulted in overlapping categories with wide VE ranges: 8.8%-78.9% in children aged <24 months; 1.6%-53.3% in children aged >24 months; and 12.6%-84.6% in children from mixed age subgroups (data not shown). Within age subgroups VE varied by types of outcome and study designs but the numbers were too small to allow further stratification.

VE for pneumonia caused by vaccine-type serotypes [15] were not considered in this review as effectiveness estimates for pneumonia were pooled together with those for bacteremia.

All three studies that assessed PCV impact on pneumonia mortality [22–24] showed substantial decline in rates after PCV introduction (Table 2; Fig 2). However, 2 of these studies reported very wide confidence intervals [22, 24], and one reported non-significant estimates [23]. Two studies also assessed pneumonia mortality as a secondary outcome: Diaz et al. [22] estimated PCV-10 VE for all-cause mortality at 38.8% (95% CI, 23.7%-44.3%) in a nested case control study; Becker-Dreps [21] estimated PCV-13 VE at 33% (95% CI 20%-43%), acknowledging that the number of pneumonia related deaths was too small to explain the reported reduction in infant mortality (138/10,000 child-years).

Analysis of effectiveness for combinations of dosing schedules and catch-up doses was not performed, due to the existing methodological variation among studies, already referred to.

### PCV effectiveness on meningitis hospitalizations and deaths

Five studies addressed PCV effectiveness against pneumococcal meningitis, all of which were conducted in Brazil, where PCV-10 is used. They comprised four before-after [19, 20, 41, 42], and one case-control study [15]. Hospitalization rates before intervention reported in the before–after studies were higher among younger children, varying from a lower 0.83/100,000 in children aged 2–3 years [20] to 14.85/100,000 in infants <1 year old [19]. (Table 3)

**Table 3:** Characteristics of studies reviewed with pneumococcal meningitis as endpoint.

First Author, year	Country	Vaccine	Study design	Case definition	Data source	Age groups*	Years of baseline data	Baseline measure (Rates p. 100,000)	Years of post PCV introduction data	Percent change/effectiveness	Statistical Significance (95% CI or p-value)
Domingues, 2014 [15, 43], Verani, 2015[16]	Brazil	PCV-10	Case-control	Pneumococcal meningitis	Cases: National laboratory surveillance; Controls: National birth registry	2m–53.1m	2010^#a^	158 cases and 1,219 controls	2.7^#b^	87·7*	61·4–96·1*
Grando, 2015 [20]	Brazil	PCV-10	Before-after	Pneumococcal meningitis	National passive surveillance system	<12m	2007–2009	7.38	2	36.6	NR
						12-23m		2.14		61.2	NR
						24-36m		0.83		13.3	NR
				Pneumococcal meningitis deaths		<12m		3.47		65.1	NR
						12-23m		0.63		56.8	NR
						24-36m		0.36		55.4	NR
Hirose, 2015 [19]	Brazil	PCV-10	Before-after	Pneumococcal meningitis	National passive surveillance system	<12m	1998–2009	14.85	2	62.8	<0.001
						12-23m		1.86		51.6	<0.001
						0-23m		6.21		59.9	<0.001
				Pneumococcal meningitis deaths		<12m		4.59		77.3	<0.001
						12-23m		0.57		68.4	<0.001
						0-23m		1.92		75.5	<0.001
Azevedo, 2015 [41] POSTER ONLY	Brazil	PCV-10	Before-after	Pneumococcal meningitis	Hospital based surveillance	0–2 yrs	2008–2010	4.23	3	48	-9–75
				VT-PCV-13 Pneumococcal meningitis		0–2 yrs				77	20–94
Liphaus, 2012 [42] POSTER ONLY	Brazil	PCV-10	Before-after	Pneumococcal meningitis	National passive surveillance system	<24m	2001–2009	10.2	1	50	p<0.001

* Age groups with results of interest for this study

#a: Start of case detection

#b: Duration of follow-up/case detection

Considering all reports on PCV-10 effectiveness regardless of age group, VE point estimates varied from 13.3% to 87.7% (Table 3). The highest reported VE was 87.7% against meningitis caused by serotypes included in PCV-10 in children aged <5 with an age-appropriate PCV-10 schedule [15].

Most studies reported effectiveness against pneumococcal meningitis of all serotypes for <12m, 12-23m, and younger than 2 years. Two studies reported on VE for children < 12 months of age, ranging from 36.6% [20] to 62.8% [19]. Lower VE effectiveness was reported for children aged 24-36m (13.3%) [20]. Effectiveness estimates ranged from 48% to 59.9% among studies reporting on children < 2 years of age [19, 41, 42].

**Fig 3:**
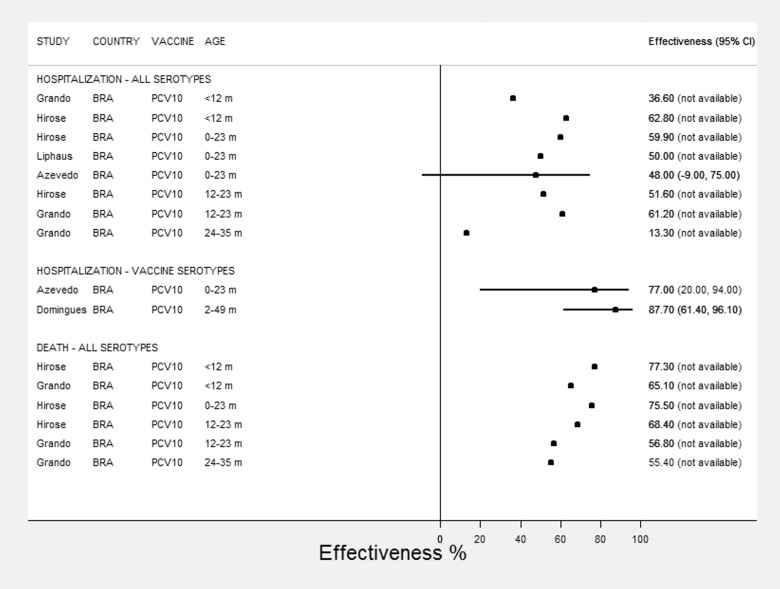
Vaccine effectiveness* against meningitis, by vaccine, group of age, vaccine serotypes and all serotypes, and hospitalization or death. * 95% confidence intervals were not reported by several of the studies and were not plotted. Country: BRA (Brazil).

As showed in Fig 3, higher effectiveness on pneumococcal meningitis hospitalizations and deaths are reported for children < 12 months of age. One study considered individuals aged 5 years and more (including adults and elderly) as a comparator group of individuals not targeted by PCV [41]. Data from a reference hospital showed small and non-significant decrease in pneumococcal meningitis in this age group, as opposed to a significant decrease of 48% for overall and 77% for vaccine-type pneumococcal meningitis in children aged <2years three years after PCV introduction. As observed in Table 3 and Fig 3, most studies reporting on vaccine effectiveness against meningitis did not report 95% confidence intervals for the estimated effectiveness measure.

Two studies addressed pneumococcal meningitis mortality [19, 20] and reported similarly high VE estimates ranging from 65–77.3% in children < 12 months to 56.8–68.4% in children aged 12–23 months.

Estimates of serotype-specific PCV-10 effectiveness against meningitis were reported by some authors, mainly as proportion of cases before and after vaccine introduction. In addition, most of such studies reported on a small number of cases with serotype data. In Paraná State, Brazil, Hirose et al. [19] reported on number of cases of pneumococcal meningitis due to PCV-10 serotypes in children less than 2 years old before and after PCV introduction. The proportion of cases due to PCV-10 serotypes was reduced from 76% of 187 cases in pre-vaccine period to 47% of 15 cases in the post-vaccine period. In a reference hospital in Salvador, Brazil [41] a 73% reduction in PCV-10 serotype cases was observed in 2011–2013, for serotypes 14 (10 cases in the pre PCV to 2 cases in the post PCV period), 19F (4 to 1) and 6B (5 to 2). Cases by 18C and 9V did not occur during post-PCV-10 period. Serotype 19A was isolated in only 3 cases before and 1 after PCV-10.

### PCV effectiveness on Invasive Pneumococcal Disease (IPD) hospitalizations

Four studies addressed invasive pneumococcal disease (IPD) hospitalizations [15, 32, 44, 45], none of which analyzed IPD mortality. Reported baseline IPD rates ranged from 3.9 (predicted rates based on time-series modelling for children aged 2–4 years) to 68.7 (children <2 years old in Uruguay) per 100,000 children (Table 4).

**Table 4:** Characteristics of studies reviewed with invasive pneumococcal disease as endpoint.

Author, year	Country	Vaccine	Study design	Case definition	Data source	Age * groups	Years of baseline data	Baseline measure (Rates p. 100,000)	Years of post PCV introduction data	Percent change/ effectiveness	Statistical Significance (95% CI or p-value)
Valenzuela, 2014 [45], ISPC, 2015 [46]	Chile	PCV-10	Interrupted time series	Spn^$^ isolated from normally sterile fluids	National Reference Laboratory	0–35m	2007–2010	24.6	2012	56.9	Not available
Garcia Gabarrot, 2014 [44]	Uruguay	PCV-13	Before-after	Spn isolated from normally sterile fluids	National Reference Laboratory	<24m	2003–2007	68.7; 24.8^#^	2009–2012	66.0; 75.0^#^	46–79; 39–90^#^
						24–60m	2003–2007	23.8; 16.0^#^	2009–2012	57.0; 56.0^#^	9–79; -6.3–82.0^#^
Domingues, 2014 [15, 43] Verani, 2015 [16]	Brazil	PCV-10	Case control	Spn isolated from normally sterile fluids	Cases: National laboratory surveillance; Controls: National birth registry	2–53.1m	2010^#a^	316 cases and 1,219 controls	2.7^#b^	83.8^#^	65.9–92.3^#^
Andrade, 2015 [47] 2016[32]	Brazil	PCV-10	Interrupted time series	Spn isolated from normally sterile fluids	National Notifiable Diseases Surveillance System; National reference laboratory	2–23m	Jan. 2008—Dec. 2009	20.9^§^	Jan. 2011 Dec. 2013	44.2	15.8–72.5
						2–11m		29.2^§^		34.7	10.4–58.9
						12–23m		13.8^§^		61.1	39.6–82.7
						24–60m		3.9^§^		-14.7	-115.1–85.7

* Age groups with results of interest for this study

# VT-PCV-13

#a: Start of case detection

#b: Duration of follow-up/case detection

§ Predicted rates based on time-series modeling

^$^ *S*. *pneumonia*

^**&**^ Verani et al. [16] reports similar VE estimates. As the authors report on the same study using different data analysis, results for Verani et al. [16] are not included in the table.

**Fig 4:**
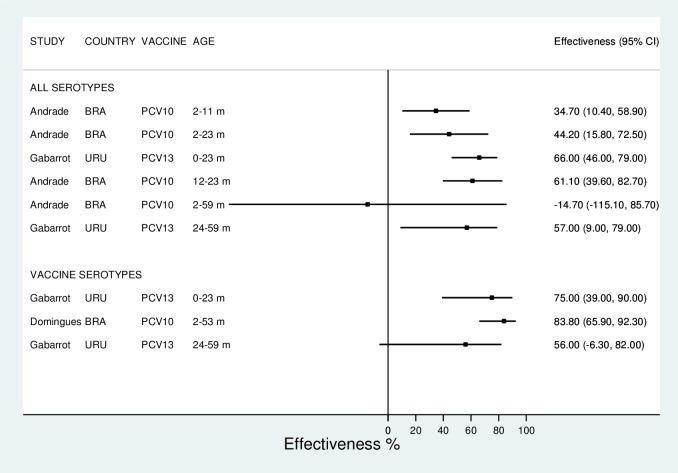
Vaccine effectiveness (%, 95% confidence interval*) against invasive bacterial disease, by vaccine, group of age, vaccine serotypes and all serotypes, and hospitalization or death. *Study by Valenzuela et al. [45] did not report confidence interval and was not plotted. Countries: BRA (Brazil); URU (Uruguay).

Reported VE against all-type IPD was generally high regardless of age, ranging from -14.7% to 66.0% (Table 4, Fig 4). Disregarding the outlier estimate by Andrade et al. [32], VE varied from 34.7% to 66.0%. Effectiveness was even higher for vaccine-types IPD, as reported by Domingues et al. [15] in a case-control study in Brazil, and Garcia Gabarrot et al. [44] in a before-after study in Uruguay (Table 4; Fig 4). Results from Domingues et al. [15] were corroborated by Verani et al. [16], based on the same study but with an indirect cohort design, which reported an adjusted VE of an age-appropriate PCV-10 schedule against type-specific pneumococcal IPD of 73.9% (95%CI 41.9%-88.3%), and 72.8% (95%CI 44.1%-86.7%), respectively, for children with up-to-date schedule, and one or more PCV doses. VE against vaccine-related types was 64.8% (95%CI 15.3%-85.4%), and 61.3% (95%CI 14.5%-82.5%), respectively, for children with up-to-date schedule, and one or more PCV doses [16].

Both interrupted time series studies conducted in Chile [45] and Brazil [32] showed a decreasing trend after PCV introduction in children aged <2 years contrasting with non-decreasing trend in rates in age groups not targeted by PCV. That resulted in consistently high VE in both studies (Table 4).

Estimates of PCV-10 effectiveness against IPD caused by individual serotypes were reported by some authors, but were mostly based on the comparison of the number of cases when comparing pre- and post-vaccine periods. A study in Chile [45] reported important decrease in serotype specific pneumococcal IPD after PCV-10 introduction in children under 2 years of age, particularly for serotypes 4 (5 cases in the pre-PCV to 0 cases in the post-PCV period), 19F (15 to 1), 23F (11 to 1), 14 (74 to 14), 6B (20 to 6), 18C (12 to 5), and 1 (11 to 2). Serotype 19A cases decreased from 13 to 8. Similarly, in Uruguay [44], important reductions of serotype specific IPD cases were reported after PCV-13 introduction in children aged < 5 years, for serotype 14, and serotype 5. Small decreases were observed for the other PCV-13 serogroups, except for serogroup 3 and 4, which showed modest increase, and 19A, which did not change substantially.

Two studies conducted in Brazil [15, 32] estimated VE for specific serotypes. In a case-control study, Domingues et al. [15] reported VE of 87.7% (95% CI 60%.8–96.1%) for serotype 14 (72 cases), 82.8% (95% CI 23.8%-96.1%) for 6B (32 cases) and 82.2% (95% CI 10.7%-96.4%) for 19A (26 cases). For serotypes 3, 6A and 23F, the effectiveness was not statistically significant. Effectiveness against IPD due to PCV-7 serotypes was 83.2% (95% CI 64.7%-92.1%). No cases of disease due to serotypes 1 or 5, and only one due to 7F, were enrolled.

Andrade et al. [32] by means of a time series analysis, found that, overall, PCV-10 type IPD declined 41.3% (p-value <0.001), mostly in children aged 2–23 months, while PCV-13 minus PCV-10 types increased by 62.8% in all age groups (p-value <0.001). This increase was mostly significant in children under 5-year of age.

### Risk of bias assessment

An inventory of potential for bias was detailed for all studies included in the review (S1 and S2 Tables). Of three studies based on individual data [15, 18, 22], one cohort study on PCV-13 did not address potential selection bias and confounding, and presented only a crude measure of association [18], indicating an unexpected high VE for consolidated pneumonia. In the case-control study by Domingues et al. [15], IPD was defined by the detection of *S*. *pneumoniae*, which enhanced specificity of case definition. However, the authors acknowledged that case ascertainment took place in a small number of hospitals with the laboratory capacity for pneumococcal identification. Moreover, case detection of IPD via surveillance system resulted in over-representation of meningitis, despite being the least common invasive syndrome. Selection of controls is a major challenge and there is no “ideal” control group for case-control studies. Neighborhood controls appeared a reasonable and efficient approach in that study conducted in a developing country [48, 49]. A study by Verani et al. [16], ancillary to the case-control study, used the “indirect cohort method” to control for biases in ascertainment between cases and controls and obtained results similar to the main study. Exposure (vaccination status) was ascertained from written documentation (vaccination cards). This source was considered reliable and allowed the classification as up-to-date for PCV-10 if the number of valid doses was greater than or equal to the number recommended for the age at hospital admission or reference date. Confounding was addressed by matching on age and by multivariate analysis of major confounders, but residual confounding was acknowledged by the authors. Finally, the case-control study by Diaz et al. [22] was nested in a birth cohort and thus protected against selection bias. Nonetheless, children from the year before introduction of PCV-10 were mostly all unvaccinated, which might have led to misclassification of vaccination status.

Before-after and ITS were the most common study designs in this review (11 studies) (Tables 2, 3 and 4), which was not surprising, as nationwide implementation of PCV in public funded immunization programs made non-exposed individuals rare and special. But those study designs are inherently vulnerable to aggregation bias, and to confounding by epidemiological and health care setting changes concomitant to vaccination. Among ITS and before-after studies, two [28, 30] presented data on potential confounders. Five studies [21, 23, 25–27, 32] included other diseases without plausible association to PCV as a comparator, and in two of them estimates of effectiveness against a pneumococcal disease were “adjusted” for the change in the comparator. In 5 studies [17, 21, 41, 44, 45] age groups not targeted by PCV were analyzed either as comparators or to assess the indirect effect of the vaccine. Two ITS studies of pneumonia used proper seasonality modeling and time trend analysis (one clinical pneumonia [25] and one consolidated pneumonia [21] with inpatients and outpatients pooled together) but had short post-vaccination periods (1 year). Short periods of observation added to the limitations of some studies. Pre-vaccination periods ranged from 2 to 12 years (median: 4 years), although one study based its effectiveness assessment on rates of the year before PCV introduction [29]. Most studies [20, 21, 24, 25, 27, 30–32, 41, 42, 44, 45] considered a transition period (usually, the year of PCV introduction), which were excluded from analysis by some, or included either in pre-vaccination or post-vaccination by others.

Selection bias was an issue rarely addressed in the studies even in those based on passive surveillance, hospital and laboratory surveillance, or sentinel surveillance systems. Only one of them acknowledged the changed approach to diagnosis engendered by PCV implementation [32].

Vaccination coverage was presented in ten studies [17, 19, 20, 24, 25, 27, 28, 30, 32, 44], for descriptive purposes only, except one [25] study in which discrepancies of effectiveness across cities were attributed to the proportion of vaccinated subjects.

## Discussion

To our knowledge this is the first systematic review of the impact and effectiveness of the 2 current commercially available pneumococcal conjugate vaccines (PCV-10 and PCV-13) in LAC countries. The thorough review of the literature allowed assessment of PCV impact and effectiveness on the most relevant clinical syndromes of pneumococcal disease, which lead to hospitalization and mortality in children under 5 years old. In this review, pneumonia was the most frequently targeted clinical presentation by the studies, which showed high incidence rates. Studies in this review showed that PCV had both direct and indirect effects on the three clinical syndromes most relevant for hospitalization, in all different age-groups, schedules, countries, and study designs, except for very few instances in untargeted age groups [50]. A positive impact of PCV-10 was also shown in a review of studies conducted in Brazil on hospitalizations and deaths from pneumonia and IPD [51].

The studies selected in our review were carried out in countries using the vaccination schedule 2+1, except Brazil which used 3+1 and Nicaragua, 3+0[8]. Brazil has since switched to a 2+1 schedule, which is also currently used in all countries in the Region where PCV has been implemented, except for 4 countries which are supported by GAVI. A recent systematic review showed that all schedules mentioned above reduced clinical and radiologically confirmed pneumonia [52, 53]. Therefore we decided to analyze the impact and effectiveness studies without any distinction of vaccine schedule established in the countries.

We selected pneumonia and IPD hospitalizations and deaths as study outcomes, which are relevant in terms of disease burden and severity, and for which there are available data sources for impact assessment. These are the most commonly measured disease outcomes in countries with PCV impact studies [9].

A variety of case definitions and endpoints for pneumonia assessment were used in the included studies. It is known that the sensitivity of pneumonia diagnosis and the estimated effectiveness of PCV on pneumonias vary according to the endpoint and case definition considered [54]. This affected data analysis and synthesis, by which results were analyzed by grouping studies with similar pneumonia endpoints. We observed higher VE when X-ray confirmed pneumonia was considered as opposed to clinical pneumonia, which considered generic clinical endpoints or diagnosis as coded by ICD-10 codes. Markedly high PCV-13 VE estimates were disclosed by two studies included in this review, in which bias and confounding had not been properly managed [18, 30]. This finding of our review is consistent with the literature where that specific end points and case definitions showed a more accurate VE on pneumonias due to pneumococcus. Moreover nonspecific and generic endpoints presented lower VE since these diagnoses likely include other pneumonias caused by pathogens other than pneumococcus [52, 53].

Four of the 12 studies that evaluated pneumonia hospitalizations considered combined endpoint of pneumonia inpatients and outpatients [24, 29–31]. These studies reported higher effectiveness rates when compared to studies assessing pneumonia inpatients only. Whereas some authors describe larger effects of PCV on pneumonia hospitalization when compared to ambulatory visits [55], selected studies have reported the opposite [56, 57]. As such, considering that the expected effectiveness could be different when including outpatients in the outcome of interest (in addition to hospitalized patients with pneumonia), we opted to conduct sensitivity analysis excluding these studies [24, 29–31], which resulted in more accurate VE estimates with a smaller range of values.

Effectiveness estimates were consistently high for meningitis and IPD when compared to other outcomes, reaching 56.8–83.8%. Other authors have found similar findings, as reported by a recent systematic review on the impact of PCV in pediatric older children in low and middle income countries [58]. This is likely due to high specificity of laboratory confirmed pneumococcal meningitis and IPD, as reported by studies conducted in high-income countries [59, 60].

Several studies reported on serotype-specific PCV effectiveness, and some authors acknowledged the small number of type-specific IPD cases and low representativeness of reported cases as an important limitation to demonstrating type specific VE [32, 45]. As expected, data on specific serotypes in studies included in this review were scarce as serotype was not one of the outcomes targeted by this review. The available data did not indicate that enough cases of serotypes 3, 6A and 19A had been averted to allow PCV-13 to show any advantage. Moreover, as fluctuations in the frequency of the serotypes can occur without selective pressure of vaccines, and considering limitations in study design and small number of cases, it is not possible to attribute increases in non-vaccine serotypes to the reduction in vaccine-type circulation in a vaccinated population as pointed out by Hirose et al. [19]. Reduction of carriage is fundamental to determine indirect and direct effects of pneumococcal vaccination with conjugate vaccines and it was highlighted in a systematic review where the reduction of risk on IPD due to 19A was discussed [61]. Additionally for robust conclusions it is important that countries implement surveillance, at least, to monitor the frequency of vaccine-type and non-vaccine type invasive pneumococcal disease in different age groups and for identification of factors influencing serotype distribution. This is crucial to allow vaccine design, implementation and continued effective control of pneumococcal disease [62].

Our findings indicate higher VE for all study outcomes in selected age groups (ie 12–23 months). This is likely a result of the fact that children in this age group had the opportunity to have completed the vaccination schedule recently. Nonetheless, as the overall disease burden is higher in younger children, impact as total burden of disease averted was most significant in younger age groups (ie <12 months), as reported in a global review of pneumococcal disease burden by age and region [63].

Issues related to study design were a major concern in this review. Most studies study analyzed secondary data, and all but two studies of pneumonia had a before-after or an interrupted time-series (ITS) approach. The assessment of vaccination through ecologic study design using aggregate data, such as ITS and before-after studies, provide impact measures that combined direct effects, related to individual protection from immune response, and indirect effects including non-vaccinated subjects who benefitted from reduced circulation of *S*. *pneumoniae* achieved with high vaccine coverage [64]. On the other hand, effectiveness measurements based on observational study designs, such as cohort and case-control studies, estimated the proportion of cases prevented in vaccinated subjects that were attributable to vaccination excluding indirect effects. A recent study describing methods and challenges for impact assessment of vaccination in LAC region reported a significant increase in the number of studies on pneumococcal vaccine impact [65]. As other authors have reported in developed countries [64], several are the methodological challenges faced when assessing vaccination impact, particularly considering PCV.

As reported in the literature [65], our results shows that most studies on impact of public health interventions used secondary data from health information systems, surveillance systems, and others sources, while few studies used primary data. As hospitalization and mortality outcomes are the most relevant outcomes of interest, it is expected that secondary data are the most used data sources. Data limitations inherent to health systems databases such as representativeness, completeness and reliability are thus present. As such, potential confounding and biases must be minimized in study design and analyses, or taken into account during result interpretation, following available recommendations [64].

This study has several limitations that are worth mentioning. While the strength of this analysis is to provide a wealth of information on the heterogeneity of the vaccine impact and effectiveness as well as on the methodological quality of the studies, there are some limitations. It was not possible to perform a meta-analysis which could allow us to estimate a common measure since we found an important heterogeneity on study designs, end points, and age group stratification within studies included in this analysis. Only studies from six countries were included in the final analysis. No studies from the Caribbean countries met the inclusion criteria for this study. The small number of countries investigated could affect the comprehensive understanding about the vaccine impact and effectiveness in Latin American countries.

Finally, potential publication bias, resulting in under publication of studies with negative results has to be considered when interpreting these results. We believe that the extensive literature search strategy and sources in our study contributed to reduce publication bias. Nonetheless, it is unclear to what extent and impact that selective publication of favorable results may have had in this review. Five of the 22 studies were funded by the industry, and 6 others had co-authors with some link to vaccine manufacturers. Nine studies were sponsored by governmental and/or international organizations and two did not disclose sponsorship. As shown in previous research [66], sponsors (including vaccine manufacturers and national immunization authorities) of studies included in this review may have contributed to give higher and earlier visibility to “positive” results. A time lag bias (favorable results published earlier) is also plausible, given that PCV introduction in national immunization programs in Latin American countries has started in 2008 and several studies have been identified in more recent years.

No studies in this review have compared the effectiveness of PCV-10 and PCV-13 directly, and thus, only indirect comparisons were possible. Considering the outcomes studied and the available evidence to date, we found no evidence of the superiority of one vaccine over the other with regards to impact and effectiveness on hospitalization reduction in children under 5 years old. Considering the inclusion criteria established in this study there is insufficient evidence so far to compare the impact and effectiveness of both vaccines with regards to mortality. Studies directly comparing the effect of PCV-10 and PCV-13 in developed countries have demonstrated similar effectiveness with different schedules on pneumonia and IPD hospitalizations [67, 68].

Currently PCV is one of the most expensive vaccines recommended by PAHO and WHO, accounting 75% of the total vaccine cost of immunizing a child in the majority of LAC countries [69]. It is crucial for policy makers to consider the affordable vaccine price whether they decide to keep the vaccines or introduce them into the national immunization programs. The available body of evidence included in this review ratifies the value of pneumococcal conjugate vaccines in the national EPI as a public health intervention, given the fact that these vaccines lead to a substantial reduction on hospitalization and mortality due to IPD, pneumonias, and meningitis in children.

## Supporting Information

S1 Appendix Protocol registered in PROSPERO (PDF)

S2 Appendix PRISMA checklist (DOC)

S3 Appendix Search strategies (PDF)

S4 Appendix Supplementary search strategies (PDF)

S5 Appendix Excluded articles and reasons for exclusion (PDF)

S1 Table Risk of bias assessment of pneumonias end point. (DOCX)

S2 Table Risk of bias assessment of pneumococcal meningitis endpoint. (DOCX)
