# Supplementary material for: Impact and Effectiveness of 10 and 13-Valent Pneumococcal Conjugate Vaccines on Hospitalization and Mortality in Children Aged Less than 5 Years in Latin American Countries: A Systematic Review
Source: PLoS One. 2016 Dec 12;11(12):e0166736. doi: 10.1371/journal.pone.0166736 (PMC5152835; doi:10.1371/journal.pone.0166736)
Supplement: S1 Appendix — (PDF) [file pone.0166736.s001.pdf]

Impact and effectiveness of 10-valent and 13-valent pneumococcal conjugate vaccines on morbidity and mortality in children aged less than 5 years in Latin America and Caribbean countries: a systematic review

Luiz Antonio Bastos Camacho, Lucia Helena Oliveira, Martha Silvia Martinez-Silveira, Cristiana Maria Toscano, Evandro Freire Coutinho

Citation

Luiz Antonio Bastos Camacho, Lucia Helena Oliveira, Martha Silvia Martinez-Silveira, Cristiana Maria Toscano, Evandro Freire Coutinho. Impact and effectiveness of 10-valent and 13-valent pneumococcal conjugate vaccines on morbidity and mortality in children aged less than 5 years in Latin America and Caribbean countries: a systematic review. PROSPERO 2016:CRD42016032693 Available from
http://www.crd.york.ac.uk/PROSPERO_REBRANDING/display_record.asp?ID=CRD42016032693

Review question(s)

What is the impact and effectiveness of 10-valent and 13-valent pneumococcal conjugate vaccines on hospitalizations and mortality from pneumococcal invasive disease, pneumonia, meningitis and sepsis in children aged less than 5 years in Latin America and Caribbean (LAC) countries?

Searches

PubMed (MEDLINE), Scopus, Lilacs, Cochrane Central Register of Controlled Trials (CENTRAL), Web of Science, congress and conference proceedings and annals, theses and dissertations, Scholar Google; handsearching from reference lists of included studies; contact with authors of included studies and with experts, vaccine manufacturers and associations related to the topic.

Types of study to be included

The review will encompass experimental and observational studies, with design features indicating longitudinal comparisons involving vaccination status. They include, but are not limited to, randomized trials (including cluster randomized trials), controlled non-randomized clinical trials and quasi-experimental studies, interrupted time series studies with at least three data points before and after the intervention, controlled before-after studies, prospective and retrospective comparative cohort studies, and case-control or nested case-control studies. We will include studies that directly compare the impact of PCV-10 and PCV-13 and studies that assess the impact of each one of those vaccines separately. Cluster randomized, cluster non-randomized and controlled before-after studies will be included only if there are at least two intervention sites and two control sites. Cross-sectional studies, case series, and case reports will not be considered for review.

Condition or domain being studied

Pneumococcal (Streptococcus pneumoniae) disease comprises major clinical syndromes, e.g., pneumonia, bacteremia and meningitis, and milder illnesses, such as sinusitis and otitis media. All age groups are affected but most of the burden falls in infants, young children and the elderly (Centers for Disease Control and Prevention 2015). Bacteremia is the most common invasive clinical presentation of pneumococcal infection in children aged 2 years or less, whereas pneumonia (non invasive disease) is the most common clinical presentation in adults. Invasive pneumococcal disease (IPD) implies the isolation of pneumococci from normally sterile body sites, such as the blood stream, or those secondary to blood stream spread, e.g. meningitis or septic arthritis (World Health Organization 2012).

Participants/ population

Healthy children (exclude studies targeting specifically children with sickle cell disease, HIV-infection or conditions known to affect immune response) aged less than five years (or with most participants in this age group), both sexes, from Latin America e Caribbean countries. Children aged 0-4 years are the most likely to benefit from vaccine against pneumococcus.

Intervention(s), exposure(s)

The study will focus on 10-valent and 13-valent pneumococcal conjugate vaccines in any immunization scheme (e.g., two or three doses in the first year of life, with or without a booster in the second year of life, with or without "catch-up" for children 12-23 months old).

Co-interventions of interest will be:

(1) other vaccines administered on the same day or within a few days (specified) before or after PCV; and

(2) other interventions indicated in the article or report.

Comparator(s)/ control

We will consider studies that compare PCV-13 with PCV-10, and studies that compare PCV-13 or PCV-10 with other interventions e.g., PCV-7, 23-valent pneumococcal polysaccharide vaccines (PPV-23) and non intervention. Data on co-interventions will also be ascertained, analogous to those indicated for the intervention group.

Context

The setting of administration (e.g., primary health care units, special vaccination rooms in remote areas etc.) will be abstracted as formatted in the article. Also of interest will be the data on the quality of the intervention (e.g., (quality of staff training for vaccine delivery, cold chain etc.) as assessed by study authors, others or by the reviewer.

Outcome(s)

Primary outcomes

The outcomes of interest are deaths or hospitalizations for invasive pneumococcal disease, pneumonia (defined by X-ray), meningitis and sepsis, excluding nosocomial infections. As disease definitions may vary in individual studies, outcome data will be extracted as defined by the authors in included studies. Outcome definitions will be refined on the basis of included studies. Timing of outcome (time after the second dose) and time of follow-up will also be assessed.

Secondary outcomes

Secondary outcomes, such as serotype specific disease, adverse events, immunogenicity (antibody levels) and nasopharyngeal carriage, will be considered accessory and reviewed to provide complementary information.

Data extraction, (selection and coding)

Selection of studies:

Titles and abstracts of identified citations will be screened by two independent researchers, who will not be blind to the journal titles or to the study authors or institutions. Screening of citations for inclusion in an SR will follow a two-stage approach: (1) screening of titles and abstracts against the inclusion criteria; and (2) screening of full-text papers passing the first screen. Screening will categorize the articles in one of the following categories: potentially eligible, related reviews, related references, unclear, and excluded. Two independent reviewers will assess the text of complete reports for all titles that appear to be eligible (and those for which eligibility is uncertain) to evaluate whether they met the inclusion criteria. Reasons for excluding studies will be recorded. Studies on whose eligibility reviewers disagree, either in screening or in the full-text review, will be discussed or assessed by a third reviewer. Contact with authors of original studies may be conducted when required by uncertainties or difficulties in decision. Inter-rater agreement (proportion agreement and Kappa statistic) will be assessed. The process will be pilot tested on a subsample of 10 articles.

To avoid multiple counting from reports of the same study we will compare publications that share some of the authors, time period, places, and other elements. Data from multiple reports of the same study will be extracted from all reports into a single form and will be collated to address the research question of this review.

Data extraction and management:

Extracting data from reports will be done independently, in duplicate, using an abstraction form (evidence table) developed specifically for this systematic review (Appendix). A pilot test of the data abstraction form and the review

process will be conducted in 10 articles before starting the review. Another pilot test will be conducted if major changes are made to the forms. Disagreements between reviewers will be assessed and the major sources of disagreement discussed to revise the process.

Items for data extraction will include author, country, contact details, type and source of financial support and publication status from reports, year of publication, study design, trial size, duration of follow-up, demographic information (average age, gender, ethnicity); number of intervention groups and intervention details (generic and the trade name of the vaccines; vaccination schedule; number of doses; changes in vaccine type); all reported outcomes (preferably from intention to treat analysis); outcome definition, secondary outcomes; setting; diagnostic criteria; comorbidity; sequence generation; allocation sequence concealment; blinding; results with summary data for each comparison group. In studies with multiple treatment groups the groups from multiple arms will be combined into a single group. Eligibility for review and reason for exclusion will be indicated.

Risk of bias (quality) assessment

Individual studies will be appraised by using the markers of validity for randomized trials in the Cochrane Risk of Bias tool (Higgins & Green, 2008), which include appropriate generation of random allocation sequence; concealment of the allocation sequence; blinding of participants, health care providers, data collectors, and outcome adjudicators; and proportion of patients lost to follow-up. Taking those six domains as reference, the risk of bias will be judged on the basis of extracted information and rated as 'high risk' or 'low risk'. If the study report does not provide sufficient details the risk of bias will be considered 'unclear' and study investigators will be contacted for more information.

Non-randomized studies will be assessed considering the data elements included in the Newcastle-Ottawa scale (NOS) to address potential sources of bias in cohort and case-control studies:

(1) For studies with comparison groups defined by outcome: case definition and gathering; approach to selection of controls; and ascertainment of exposure;

(2) For studies with comparison groups defined by exposure/intervention: baseline characteristics; selective participation, withdrawal and loss to follow-up; approach to outcome ascertainment;

Differential reporting of study findings will be checked in all study designs.

Potential confounders of the association between vaccination and the outcomes of interest include: age, sex, race/ethnicity, socioeconomic status (income, literacy, occupation of parents etc.); urban/rural residence and others considered by the authors. f The design features and analytical approaches set out by researchers to control confounding will be searched and the likelihood of residual confounding analyzed. The risk of bias will be analyzed as a source of heterogeneity.

Time-series studies will be assessed with a modified version of Ramsey et al´s items (Ramsey et al., 2003).

A third reviewer will be consulted when disagreements between independent reviewers on the risk of bias are not resolved by discussion.

Strategy for data synthesis

The numbers of studies throughout the process of study selection will be represented in a flowchart. A table with descriptive information for each study will be produced. The information will be presented by type of study design, e.g., report results for randomized controlled trials, and from non-randomized trials and non-experimental studies, with PCV-10 and PCV-13 in comparison groups or separately. The study will report on studies of any level of risk of bias in their analyses.

Meta-analysis will be considered in this review as a means to improve precision in estimates of the impact PCV-10 and PCV-13 and increasing the chance of detecting statistically significant differences between them. It will also allow us to address issues not raised by individual studies, such as, difference in impact between PCV-10 and PCV-13 and variation of impact across immunization schedules.

The heterogeneity of immunization effects on hospitalizations and deaths from pneumococcal invasive disease, pneumonia, meningitis and sepsis across studies will be assessed by visual examination of the forest plot, quantified using the Higgins I-squared statistic and tested using the chi square test. If high levels of heterogeneity among the trials exist (I-squared >=50% or P<0.1) the study design and characteristics in the included studies will be analyzed. In addition to statistical heterogeneity, two sources of variability of results across studies will be considered: (1) diversity in characteristics of study participants, interventions (type of vaccine and immunization regimen) and outcome ascertainments; and (2) diversity of study design, conduct and quality (risk of bias).

Should meta-analysis be considered possible and sensible on the basis of analysis of heterogeneity, pooled measures of association may be calculated separately for combinations of PCV and comparators. The Mantel-Haenszel method will be used for the fixed effect model if tests of heterogeneity are not significant. Summary measurement will be plotted for subgroups of study designs and vaccine types. Statistical uncertainty will be assessed by using 95% confidence intervals around estimates. Each outcome will be combined and calculated using the Stata Statistical Software: Release 12 (College Station,TX: StataCorp LP). The random effects model will be chosen if statistical heterogeneity is observed (I-SQUARED >=50% or P<0.1). We will not conduct a meta-analysis in case the heterogeneity is also substantial in other methodological aspects.

A narrative, qualitative summary will be performed whether or not a meta-analysis has been done. A narrative synthesis will be based on information summarized in tables with the characteristics and findings of the included studies: country, year of publication, number of participants, age range, name of vaccine, immunization schedule, comparator, study design, outcomes, magnitude of effect, and confidence interval.

Analysis of subgroups or subsets
Data analysis will consider the following subgroups: study design; risk of bias (high; low); type of vaccine (PCV-10, PCV-13), vaccination schedule, number of doses (=1 dose, =2 doses), country, age, race/ethnicity (white, black, indigenous, mixed, other), residence (rural, urban), socioeconomic status (high, middle, low), and Streptococcus pneumoniae serotypes (vaccine, non-vaccine serotypes).

Dissemination plans
Report of this review will be submitted to a major journal in the field of vaccinalogy and to the Pan-American Health Organization to subsidize recommendations regarding pneumococcal vaccines.

Contact details for further information
Dr Bastos Camacho

Escola Nacional de Saude Publica-Fiocruz

Rua Leopoldo Bulhaes, 1480, sala 820. Manguinhos

Rio de Janeiro-RJ, 21041-210. Brazil

luiz.camacho@ensp.fiocruz.br

Organisational affiliation of the review
PanAmerican Health Organization

Review team
Dr Luiz Antonio Bastos Camacho, Oswaldo Cruz Foundation (Fiocruz)
Dr Lucia Helena Oliveira, PanAmerican Health Organization (PAHO)
Dr Martha Silvia Martinez-Silveira, Oswaldo Cruz Foundation (Fiocruz)
Dr Cristiana Maria Toscano, Federal University of Goiás
Dr Evandro Freire Coutinho, Oswaldo Cruz Foundation (Fiocruz)

Anticipated or actual start date
01 December 2015

Anticipated completion date
29 January 2016

Funding sources/sponsors
Sabin Vaccine Institute

Pan-American Health Organization

Conflicts of interest
None known

Language
English

Country
Brazil

Subject index terms status
Subject indexing assigned by CRD

Subject index terms
Caribbean Region; Child; Humans; Latin America; Pneumococcal Vaccines; Vaccines, Conjugate

Stage of review
Ongoing

Date of registration in PROSPERO
09 January 2016

Date of publication of this revision
09 January 2016

**Stage of review at time of this submission**	**Started**	**Completed**
Preliminary searches	Yes	Yes
Piloting of the study selection process	Yes	Yes
Formal screening of search results against eligibility criteria	Yes	No
Data extraction	No	No
Risk of bias (quality) assessment	No	No
Data analysis	No	No

PROSPERO
International prospective register of systematic reviews
The information in this record has been provided by the named contact for this review. CRD has accepted this information in good faith and registered the review in PROSPERO. CRD bears no responsibility or liability for the content of this registration record, any associated files or external websites.
