# Supplementary material for: Impact and Effectiveness of 10 and 13-Valent Pneumococcal Conjugate Vaccines on Hospitalization and Mortality in Children Aged Less than 5 Years in Latin American Countries: A Systematic Review
Source: PLoS One. 2016 Dec 12;11(12):e0166736. doi: 10.1371/journal.pone.0166736 (PMC5152835; doi:10.1371/journal.pone.0166736)
Supplement: S3 Appendix — (PDF) [file pone.0166736.s003.pdf]

S1 Appendix: Databases Search strategies

DATABASE	SEARCH STATEGY
**MEDLINE/PUBMED**<br>**Date run:**<br>Oct 20th, 2015<br>Feb 2nd, 2016	**1. CONDITION:**<br>"Pneumococcal infections"[MeSH Terms] OR "Invasive pneumococcal disease"[All Fields] OR ("pneumococcal"[All Fields] AND "infections"[All Fields]) OR "pneumococcal infections"[All Fields] OR "Pneumonia, Pneumococcal"[MeSH Terms]OR ("pneumonia"[All Fields] AND "pneumococcal"[All Fields]) OR "pneumonia pneumococcal"[All Fields] OR (("streptococcus"[MeSH Terms] OR "streptococcus"[All Fields]) AND ("pneumonia"[MeSH Terms] OR pneumonia*[All Fields])) OR “Streptococcus pneumoniae”[MeSH Terms] OR “Streptococcus pneumonia”[All Fields] OR “Streptococcus pneumoniae”[All Fields] OR "meningitis"[MeSH Terms] OR "meningitis"[All Fields] OR "meningitis, pneumococcal"[MeSH Terms] OR ("meningitis"[All Fields] AND "pneumococcal"[All Fields]) OR "pneumococcal meningitis"[All Fields] OR (Meningitis, Bacterial[MeSH Terms] OR (("meningitis"[MeSH Terms] OR "meningitis"[All Fields]) AND Bacterial[All Fields])) OR "sepsis"[MeSH Terms] OR "sepsis"[All Fields]<br>**2. INTERVENTION**<br>(("pneumococcal vaccines"[MeSH Terms] OR ("pneumococcal"[All Fields] AND "vaccines"[All Fields]) OR "pneumococcal vaccines"[All Fields] OR ("pneumococcal"[All Fields] AND "vaccine"[All Fields]) OR "pneumococcal vaccine"[All Fields] OR"13-valent pneumococcal vaccine"[Supplementary Concept] OR "13-Valent Pneumococcal Conjugate Vaccine"[TIAB] OR "13-Valent Pneumococcal Vaccine"[TIAB] OR "13 valent pneumococcal conjugate vaccine"[All Fields] OR "13 valent pneumococcal vaccine"[All Fields] OR “13-valent conjugate vaccine”[All Fields] OR “13 valent conjugate vaccine”[All Fields] OR PCV13[TIAB] OR "10-valent pneumococcal conjugate vaccine"[Supplementary Concept] OR "10-valent pneumococcal conjugate vaccine"[All Fields] OR "10 valent pneumococcal conjugate vaccine"[All Fields] OR "10-valent pneumococcal vaccine"[Supplementary Concept] OR "10-valent pneumococcal vaccine"[All Fields] OR "10 valent pneumococcal vaccine"[All Fields] OR "ten valentpcv"[All Fields] OR "pcv10"[All Fields] OR “10-valent conjugate vaccine”[All Fields] OR “7-valent pneumococcal vaccine”[All Fields] OR “7-Valent Pneumococcal Conjugate Vaccine"[All Fields] OR “7 Valent Pneumococcal Conjugate Vaccine"[All Fields] OR “7 Valent Pneumococcal Vaccine"[All Fields] OR "heptavalent pneumococcal conjugate vaccine"[Supplementary Concept] OR "heptavalent pneumococcal conjugate vaccine"[All Fields] OR

	“heptavalent pneumococcal vaccine”[All Fields] OR “7-valent conjugate vaccine”[All Fields] OR "thirteen-valent"[All Fields] OR "thirteen valent"[All Fields] OR "13-valent"[All Fields] OR "13 valent"[All Fields] OR "10 valent"[All Fields] OR "10 valent"[All Fields] OR "ten-valent"[All Fields] OR "ten valent"[All Fields] OR "7-valent"[All Fields] OR "7 valent"[All Fields] OR "seven-valent"[All Fields] OR “seven valent”[All Fields] OR "heptavalent"[All Fields])) OR ((("vaccines"[MeSH Terms] OR "vaccination"[MeSH Terms] OR "vaccination"[All Fields] OR vaccinated[All Fields] OR vaccine*[TIAB]) AND (("meningitis"[MeSH Terms] OR "meningitis"[All Fields]) OR ("sepsis"[MeSH Terms] OR "sepsis"[All Fields]) OR ("pneumonia"[MeSH Terms] OR "pneumonia"[All Fields] OR "pneumoniae"[All Fields]))) OR ("Vaccines, Conjugate"[MeSH Terms] OR (("vaccines"[MeSH Terms] OR vaccine*[All Fields]) AND Conjugate[All Fields]) OR "conjugate vaccines"[All Fields] OR "vaccines conjugate"[All Fields] AND (("meningitis"[MeSH Terms] OR "meningitis"[All Fields]) OR ("sepsis"[MeSH Terms] OR "sepsis"[All Fields]) OR ("pneumonia"[MeSH Terms] OR "pneumonia"[All Fields] OR "pneumoniae"[All Fields]))) OR (("immunisation"[All Fields] OR "immunization"[All Fields] OR "immunization programs"[MeSH Terms] OR "immunization programs"[All Fields] OR "immunization"[MeSH Terms]) AND (("meningitis"[MeSH Terms] OR "meningitis"[All Fields]) OR ("sepsis"[MeSH Terms] OR "sepsis"[All Fields]) OR ("pneumonia"[MeSH Terms] OR "pneumonia"[All Fields] OR "pneumoniae"[All Fields]))) OR ("PHiD-CV vaccine"[Supplementary Concept] OR "PHiD-CV vaccine"[All Fields] OR "prevnar"[All Fields] OR “prevenar”[All Fields] OR "synflorix"[All Fields]))<br><br>**AND**<br><br>**3.** ("hospitalisation"[TIAB] OR "hospitalization"[MeSH Terms] OR "hospitalization"[TIAB]) OR (("hospitals"[MeSH Terms] OR "hospitals"[TIAB] OR "hospital"[TIAB]) AND admission[TIAB]) OR effectiveness[TIAB] OR "mortality"[Subheading] OR "mortality"[TIAB] OR "mortality"[MeSH Terms] OR "epidemiology"[Subheading] OR morbid*[TIAB] OR "morbidity"[MeSH Terms] OR "incidence"[TIAB] OR "incidence"[MeSH Terms] OR "epidemiology"[TIAB] OR "epidemiology"[MeSH Terms] OR efficacy[TIAB] OR “effectiveness”[TIAB] OR (("vaccines"[MeSH Terms] OR "vaccines"[TIAB] OR "vaccine"[TIAB]) AND effects[All Fields]) OR (("vaccines"[MeSH Terms] OR "vaccines"[TIAB] OR "vaccine"[TIAB]) AND impact[All Fields]) OR protection[TIAB] OR benefits[TIAB] OR "prevention and control"[Subheading] OR "control"[TIAB] OR impact[TIAB]

	**AND**<br>**4. GEOGRAPHIC LIMIT**<br>Anguilla[TIAB] OR Anguilla[AD] OR "Antigua and Barbuda"[MESH] OR Antigua[TIAB] OR Antigua[AD] OR "Argentina"[MeSH Terms] OR Argentina[TIAB] OR Argentina[AD] OR Argentine[TIAB] OR Argentino[TIAB] OR Aruba[TIAB] OR Aruba[AD] OR "bahamas"[MeSH Terms] OR "bahamas"[TIAB] OR Bahamas[AD] OR "barbados"[MeSH Terms] OR "barbados"[TIAB] OR Barbados[AD] OR "belize"[MeSH Terms] OR "belize"[TIAB] OR Belize[AD] OR belize OR bonnaire OR "San Eustaquio" OR eustatius OR "chile"[MeSH Terms] OR "chile"[TIAB] OR Chile[AD] OR "Costa Rica"[MeSH Terms] OR "costa rica"[TIAB] OR "Costa Rica"[AD] OR "Cuba"[MESH] OR Cuba[TIAB] OR Cuba[AD] OR curacao OR "Dominica"[MESH] OR Dominica[TIAB] OR Dominica[AD] OR "grenada"[MeSH Terms] OR "grenada"[TIAB] OR Grenada[AD] OR granada OR guadalupe OR "Guadeloupe"[MeSH Terms] OR Guadeloupe[TIAB] OR Guadeloupe[AD] OR "Turks and Caicos Islands" OR "Virgin Islands of the United States"[All Fields] OR "united states virgin islands"[MeSH Terms] OR "Virgin Islands"[TIAB] OR "Virgin Islands"[AD] OR "jamaica"[MeSH Terms] OR "jamaica"[TIAB] OR Jamaica[AD] OR "Martinique"[MeSH Terms] OR Martinique[TIAB] OR Martinique[AD] OR "Puerto Rico"[MeSH Terms] OR "Puerto Rico"[TIAB] OR "Puerto Rico"[AD] OR "Saint Kitts and Nevis"[TIAB] OR "St. Kitts"[TIAB] OR "St. Kitts"[AD] OR "saint kitts and nevis"[MeSH Terms] OR "Saint Lucia"[MeSH Terms] OR "Saint Lucia"[TIAB] OR "Saint Lucia"[AD] OR "Santa Lucia" OR "Saint Vincent and the Grenadines"[MeSH Terms] OR "Saint Vincent and the Grenadines"[TIAB] OR "Saint Vincent and the Grenadines"[AD] OR "Saint Martin"[TIAB] OR "Saint Martin"[AD]OR "Sint Maarten"[AD] OR "Saint-Martin"[AD] OR "Saint-Martin"[TIAB] OR "suriname"[MeSH Terms] OR "surinam*"[TIAB] OR Surinam*[AD] OR "Trinidad and Tobago"[MESH] OR "Trinidad and Tobago"[TIAB] OR "Trinidad and Tobago"[AD] OR "Trinidad Tobago" OR "uruguay"[MeSH Terms] OR "uruguay"[TIAB] Uruguay[AD] OR "haiti"[MeSH Terms] OR "haiti"[TIAB] OR Haiti[AD] OR "brazil"[MeSH Terms] OR Brazil*[TIAB] OR Brazil[AD] OR Brasil[TIAB] OR Brasil[AD] OR "colombia"[MeSH Terms] OR colombia*[TIAB] OR Colombia[AD] OR dominican*[TIAB] OR Dominican*[AD] OR "Dominican Republic"[TIAB] OR "Dominican Republic"[AD] OR "El Salvador"[TIAB] OR "El Salvador"[AD] OR "guyana"[MeSH Terms] OR "guyana"[TIAB] OR Guyana[AD] OR Guiana[TIAB] OR Guiana[AD] OR "honduras"[MeSH Terms] OR "honduras"[TIAB] OR Honduras[AD] OR Hondurans[TIAB] OR "mexico"[MeSH Terms] OR mexic*[TIAB] OR Mexico[AD] OR

	"panama"[MeSH Terms] OR "panama"[TIAB] OR Panama[AD] OR "paraguay"[MeSH Terms] OR paraguay*[TIAB] OR Paraguay[AD] OR "venezuela"[MeSH Terms] OR Venezuela*[TIAB] OR Venezuela[AD] OR "bolivia"[MeSH Terms] OR Bolivia*[TIAB] OR Bolivia[AD] OR "ecuador"[MeSH Terms] OR "ecuador"[TIAB] OR Ecuador[AD] OR Equator[AD] OR Equatorian[TIAB] OR "guatemala"[MeSH Terms] OR "guatemala"[AD] OR Guatemal*[TIAB] OR "nicaragua"[MeSH Terms] OR "nicaragua"[AD] OR Nicaragua*[TIAB] OR "peru"[MeSH Terms] OR "peru"[AD] OR Peru[TIAB] OR Peruvian[TIAB] OR ("cayman"[All Fields] AND "islands"[All Fields]) OR "cayman islands"[All Fields]<br>**OR**<br>**5.“**Caribbean Region”[MeSH Terms] OR Caribbe* OR "west indies"[MeSH Terms] OR ("west"[All Fields] AND "indies"[All Fields]) OR "west indies"[All Fields] OR "montserrat"[All Fields] OR "latinamerica"[MeSH Terms] OR ("latin"[All Fields] AND "america"[All Fields]) OR "latinamerica"[All Fields] OR Antilles OR “Antillas” OR “Netherlands Antilles”[MeSH Terms] OR “Southern Cone” OR “South America” OR “South America”[MeSH Terms] OR “South American” OR “Central America” OR “Central America”[MeSH Terms] OR Centroamerica* OR “America Central” OR “America del Sur” OR Sulamerica OR Sudamerica OR Worldwide[TI] OR Global[TI]<br>**AND**<br>**6. AGE LIMIT**<br>8.("infant"[MeSH Terms] OR "infant"[All Fields] OR "child"[MeSH Terms] OR "child"[All Fields] OR "childhood"[All Fields] OR "children"[All Fields] OR "child, preschool"[MeSH Terms] OR “pediatrics"[MeSH Terms] OR "pediatrics"[TIAB] OR “paediatrics"[TIAB] OR “toddler”[TIAB)<br>**1 AND 2 AND 3 AND (4 OR5) AND 6**<br>**DATA FILTER**: FROM 2009 - 2016
**LILACS**<br>**Date run:**<br>Oct 21st, 2015<br>Feb 2nd, 2016	**1 Condition:**<br>(mh:(pneumonia pneumocócica)) OR (mh:(meningite pneumocócica)) OR (mh:(sepse)) OR (mh:(infecções pneumocócicas)) OR (tw:(pneumonia)) OR (tw:(neumonia)) OR (tw:(meningite)) OR (tw:(meningitis)) OR (tw:(sepse)) OR (tw:(sepsis)) OR (tw:(septisemia))<br>**2 Intervention:**<br>((mh:(vacinas pneumocócicas)) OR (tw:(vacina*)) OR (tw:(vacuna*)) OR (tw:(vaccine*)) OR (tw:(prevnar)) OR (tw:(prevenar)) OR (tw:(synflorix)) OR (tw:(pcv13)) OR

	(tw:(pcv10)) OR (tw:(pcv7)) OR (tw:("anti-neumocóccica")) OR (tw:("neumocóccica")) OR (tw:(antipneumocócica)) OR (tw:("7 valente")) OR (tw:("7-valente")) OR (tw:("7 valent")) OR (tw:("7-valent")) OR (tw:("vacina conjugada")) OR (tw:("13-valent")) OR (tw:("13 valent")) OR (tw:("13-valente")) OR (tw:("13 valente")) OR (tw:("10-valente")) OR (tw:("10valente")) OR (tw:("10 valent")) OR (tw:("10-valent")))<br>**AND NOT**<br>**3.** ti:(idoso* OR "adulto mayor" OR "adultos mayores" OR aged)<br>**(1 AND 2) AND NOT 3**<br>LIMITS YEAR=2009 to 2016
**SCOPUS**<br>**Date run:**<br>Oct 21st, 2015<br>Feb 2nd, 2016	1. TITLE-ABS-KEY(pneumonia OR meningitis OR septicemia OR sepsis OR "Pneumonia Pneumococcal" OR "Streptococcus pneumoniae" OR "meningitis pneumococcal")<br>2. (TITLE-ABS-KEY("pneumococcal vaccines" OR "13-valent pneumococcal vaccine" OR "13-Valent Pneumococcal Conjugate Vaccine")) OR (TITLE-ABS-KEY ("10-valent pneumococcal vaccine" OR "10-valent pneumococcal conjugate vaccine")) OR (TITLE-ABS-KEY ("7-valent pneumococcal vaccine" OR "7-valent pneumococcal conjugate vaccine")) OR (TITLE-ABS-KEY((vaccines# AND conjugate) OR synflorix OR prevnar OR prevenar)) OR (TITLE-ABS-KEY ("PCV7" OR "PCV10" OR "PCV13"))<br>3. (TITLE-ABS-KEY(hospitalization OR effectiveness OR mortality OR epidemiology OR morbidity OR incidence OR effectiveness OR (vaccine AND effect#) OR (vaccine AND impact) OR protection OR benefits OR (prevention AND control) OR impact)<br>4. (TITLE-ABS-KEY(Anguilla OR "Antigua and Barbuda" OR Antigua OR argentina OR argentin# OR aruba OR Bahamas OR Barbados OR belize OR bonnaire OR "San Eustaquio" OR eustatius OR chile#)) OR (TITLE-ABS-KEY("Costa Rica" OR cuba OR curacao OR dominica OR grenada OR Granada OR Guadalupe OR Guadeloupe OR "Turks and Caicos Islands" OR "Virgin Islands of the United States" OR "united states virgin islands" OR "Virgin Islands" OR jamaica#)) OR (TITLE-ABS-KEY(Martinique OR martinica OR "PUERTO RICO" OR "Saint Kitts and Nevis" OR "St. Kitts" OR "SANTA LUCIA" OR "Saint Vincent and the Grenadines" OR "SAINT VINCENT" OR "SAINT MAARTEN" OR surinam#)) OR (TITLE-ABS-KEY("Trinidad and Tobago" OR "TRINIDAD TOBAGO" OR uruguay# OR Haiti OR brazil# OR brasil OR colombia# OR dominican# OR "Dominican Republic" OR "EL SALVADOR" OR Guyana OR guiana)) OR (TITLE-ABS-KEY(Honduras OR Hondurans OR mexico OR

	mexic# OR panama OR Paraguay OR venezuela# OR bolivia# OR Ecuador OR equator# OR guatemal# OR nicaragua# OR peru OR Peruvian OR cayman OR "cayman islands")) OR (TITLE-ABS-KEY("Caribbean Region" OR caribbe# OR "westindies" OR Montserrat OR "latin america" OR Antilles OR antillas OR "Netherlands Antilles" OR "Southern Cone" OR "South America" OR "South American"))OR(TITLE-ABS-KEY("Central America" OR centroamerica# OR "America Central" OR "America Del Sur" OR sulamerica OR sudamerica OR worldwide OR global))<br>**1 AND 2 AND 3 AND 4**<br>LIMITS YEAR=2009 to 2016
**WEB OF SCIENCE**<br>**Date run:**<br>Oct 22nd, 2015<br>Feb 2nd, 2016<br>Index searched:<br>SCI-EXPANDED, SSCI,<br>A&HCI,<br>CPCI-S,<br>CPCI-SSH.	1. TS=(pneumonia OR pneumoniae OR meningitis OR septicemia OR sepsis OR "Pneumonia Pneumococcal" OR "Streptococcuspneumoniae" OR "meningitispneumococcal")<br>2. TS=("pneumococcal vaccines" OR (pneumococcal AND vaccines) OR "13-valent pneumococcal vaccine" OR "13-Valent Pneumococcal Conjugate Vaccine" OR "10-valent pneumococcal vaccine" OR "10-valent pneumococcal conjugate vaccine" OR "7-valent pneumococcal vaccine" OR "7-valent pneumococcal conjugate vaccine" OR “10-valent conjugate vaccine” OR “10 valent conjugate vaccine” OR “13-valent conjugate vaccine” OR “13 valent conjugate vaccine” OR (vaccine* AND conjugate) OR synflorix OR prevnar OR prevenar OR "PCV7" OR "PCV10" OR "PCV13" OR "thirteen valent” OR "13-valent" OR "13 valent" OR "10 valent" OR "10 valent" OR "ten-valent"OR "ten valent" OR "7-valent" OR "7 valent" OR "seven-valent" OR “seven valent” OR "heptavalent")<br>3. TS=(hospitalization OR effectiveness OR mortality OR epidemiology OR morbidity OR incidence OR (vaccine* AND effect*) OR (vaccine* AND impact) OR protection OR benefits OR (prevention AND control) OR impact)<br>4. TS=(anguilla OR "Antigua and Barbuda" OR antigua OR argentina OR argentin* OR aruba OR bahamas OR barbados OR belize OR bonnaire OR "San Eustaquio" OR eustatius OR chile* OR "Costa Rica" OR cuba OR curacao OR dominica OR grenada OR granada OR guadalupe OR guadeloupe OR "Turks and Caicos Islands" OR "Virgin Islands of the United States" OR "united states virgin islands" OR "Virgin Islands" OR jamaica* OR martinique OR martinica OR "Puerto Rico" OR "Saint Kitts and Nevis" OR "St. Kitts" OR "Santa Lucia" OR "Saint Vincent and the Grenadines" OR "Saint Vincent" OR "Saint Martin" OR “Sint Maarten” OR “Saint-Martin” OR surinam* OR "Trinidad and Tobago" OR "Trinidad Tobago" OR uruguay* OR haiti OR brazil* OR brasil OR colombia* OR dominican* OR "Dominican Republic" OR "EL SALVADOR" OR guyana OR guiana OR

	honduras OR hondurans OR mexico OR mexic* OR panama OR paraguay OR venezuela* OR bolivia* OR ecuador OR equator* OR guatemal* OR nicaragua* OR peru OR peruvian OR cayman OR "cayman islands" OR "Caribbean Region" OR caribbe* OR "west indies" OR montserrat OR "latinamerica" OR antilles OR antillas OR "Netherlands Antilles" OR "Southern Cone" OR "South America" OR "South American" OR "Central America" OR centroamerica* OR "America Central" OR "America Del Sur" OR sulamerica OR sudamerica)<br>**1 AND 2 AND 3 AND 4**<br>DATE LIMIT: 2009-2016
**CENTRAL**<br>**Date run:**<br>Nov 11st, 2015<br>Feb 2nd, 2016	#1 "Streptococcus pneumoniae":ti,ab,kw or "Pneumonia Pneumococcal":ti,ab,kw or "meningitis pneumococcal":ti,ab,kw or sepsis:ti,ab,kw (Word variations have been searched)<br>#2 "pneumococcal vaccines":ti,ab,kw or PCV10:ti,ab,kw or PCV13:ti,ab,kw or Pentavalent:ti,ab,kw or "13-valent pneumococcal vaccine":ti,ab,kw (Word variations have been searched)<br>#3#1 and #2<br>#4 "Child":ti,ab,kw or "infant":ti,ab,kw or "toddler":ti,ab,kw (Word variations have been searched)<br>#5 #3 and #4 Publication Year from 2009 to 2015, in Trials and Cochrane Groups
