# Supplementary material for: Impact and Effectiveness of 10 and 13-Valent Pneumococcal Conjugate Vaccines on Hospitalization and Mortality in Children Aged Less than 5 Years in Latin American Countries: A Systematic Review
Source: PLoS One. 2016 Dec 12;11(12):e0166736. doi: 10.1371/journal.pone.0166736 (PMC5152835; doi:10.1371/journal.pone.0166736)
Supplement: S4 Appendix — (PDF) [file pone.0166736.s004.pdf]

S2 Appendix: Full strategies for grey, unpublished and supplementary search

**Type of search**	**Resources accessed**
Grey literature websites searched	Grey literature report – (http://www.greylit.org)<br>SIGLE- System for Information on Grey Literature in Europe - Open Grey –(http://www.opengrey.eu)<br>Clinical Medicine NetPrints – (https://searchworks.stanford.edu)<br>Gray Literature in Health Research (http://researchguides.dml.georgetown.edu/content.php?pid=352972&sid=2887419)<br>Grey Literature in the Health Sciences (http://guides.library.upenn.edu/healthgreylit?hs=a)
Journals reviewed	Annals of Vaccines and Immunization<br>International Journal of Vaccines & Vaccination<br>International Journal of Vaccines and Immunization<br>Journal of Vaccines<br>Journal of Vaccines & Vaccination<br>Journal of Vaccines and Immunology<br>Paediatric Respiratory Reviews<br>Revista Argentina de Salud Pública<br>Revista Chilena de Salud Pública<br>Vaccine: Development and Therapy<br>World Journal of Vaccines<br>152 issues reviewed
Conference and Meetings annals or proceedings reviewed	World Congress of the World Society for Pediatric Infectious Diseases<br>Annual Meeting of the European Society Pediatric Infection Diseases<br>International Symposium on Pneumococci & Pneumococcal Diseases<br>Jornadas Nacionais de Imunização da Sociedade Brasileira de Imunizações<br>Congreso Latinoamericano de Infectología Pediátrica<br>International Congress on Pediatric Pulmonology<br>American Society of Tropical Medicine & Hygiene Annual Meeting<br>IDweek, Infection Disease Society of America<br>Symposium on New Vaccines<br>Interscience Conference on Antimicrobial Agents and Chemotherapy<br>Annual Meeting of the American Society for Microbiology<br>International Society of Vaccines Annual Global Congress
Finished and ongoing trial sites reviewed	International Standard Randomized Controlled Trial Number Registry<br>EU Clinical Trials Register<br>Clinicaltrials.gov<br>Registro Brasileiro de Ensaios Clínicos<br>Brazil Clinical Trials

	WHO International Clinical Trials Registry Platform
Manufacturer and Expert contacted	16 experts<br>2 manufacturers
Reference screening reviewed	22 Articles selected<br>41 Reviews<br>3200 references screened

Supplementary search result by type of document retrieved (January 2009 – January 2016)

Type of document	Reference screening	Hand search journals	Clinical trials search	Meeting, Congress abstract book	Expert contact, GSK/Pfizer	Grey literature database search	Total
Journal Article	10	1	0	0	7	0	18
Abstract Poster	3	0	0	50	28	0	81
Abstract Oral Presentation	6	0	0	33	50	0	89
PPT Presentation	1	0	0	0	0	0	1
Report	3	0	1	0	0	0	4
Total	23	1	1	83	85	0	193
