# Supplementary material for: Impact and Effectiveness of 10 and 13-Valent Pneumococcal Conjugate Vaccines on Hospitalization and Mortality in Children Aged Less than 5 Years in Latin American Countries: A Systematic Review
Source: PLoS One. 2016 Dec 12;11(12):e0166736. doi: 10.1371/journal.pone.0166736 (PMC5152835; doi:10.1371/journal.pone.0166736)
Supplement: S5 Appendix — (PDF) [file pone.0166736.s005.pdf]

S3 Appendix: Excluded articles and reasons for exclusion

Excluded Articles	Reasons
Tregnaghi MW, Saez-Llorens X, Lopez P, Abate H, Smith E, Posleman A, et al. Efficacy of pneumococcal nontypable Haemophilus influenzae protein D conjugate vaccine (PHiD-CV) in young Latin American children: A double-blind randomized controlled trial. PLoS Med. 2014;11(6):e1001657. doi: 10.1371/journal.pmed.1001657. PubMed PMID: 24892763.	Efficacy not impact/artificial setting
Hortal M, Estevan M, Laurani H, Iraola I, Meny M. Hospitalized children with pneumonia in Uruguay: pre and post introduction of 7 and 13-valent pneumococcal conjugated vaccines into the National Immunization Program. Vaccine. 2012;30(33):4934-8. doi: 10.1016/j.vaccine.2012.05.054. PubMed PMID: 22664222.	Before-after study; one year after PCV-13 replaced PCV-7
Abrao WM, Mello LM, Silva AS, Nunes AA. Impact of the antipneumococcal conjugate vaccine on the occurrence of infectious respiratory diseases and hospitalization rates in children. Rev Soc Bras Med Trop. 2015;48(1):44-9. doi: 10.1590/0037-8682-0007-2015. PubMed PMID: 25860463.	Does not allow data extraction
Assandri E, Amorin B, Gesuele JP, Algorta G, Pirez MC. Enfermedad neumoccocica invasora en recien nacidos, antes y despues de la vacunacion universal con vacuna conjugada 7 y 13 valente en Uruguay. Rev Chilena Infectol. 2015;32(2):167-74. doi: 10.4067/s0716-10182015000300005. PubMed PMID: 26065449.	Report on neonates
Manjarres-Posada NI, Choconta-Piraquive LA, De la Hoz-Restrepo F. Evaluating the effectiveness of conjugated pneumococcal vaccines in Colombia. Procedia Vaccinol. 2012;6:150-5. doi: 10.1016/j.provac.2012.04.020.	Only PCV-7 effectiveness evaluation
Algorta G, Machado K, Varela A, Amaya G, Assandri E, Mota MI, et al. Sertoype 1 and 5 pneumococcal pneumonia in a referral children hospital between 1998 and 2013. Uruguay. Poster session: Severe Childhood Bacterial Infections at: 9th World Conference of the World Society for Pediatric Infectious Diseases; 2015 Nov 18-21; Rio de Janeiro, Brazil, 2015 [cited 2016 Jul 7]. Abst 0619. Available from: http://wspid.kenes.com/Documents/WSPID%20All%20Abstracts.pdf	No age group stratification

Chacon-Cruz E, Alvelais-Arzamendi A, Stevanovic-Mihailovic T, Rivas-Landeros RM, Volker-Soberanes ML, Armenta-Llanes O. Reduction of pneumococcal invasive disease, disappearance of serotype 19-a and pneumococcal meningitis following 13-valent pneumococcal conjugated vaccine in Mexican children. Poster session: Controlling Pneumococcal Disease Around the Globe at: 9th International Symposium on Pneumococci and Pneumococcal Diseases; 2014 Mar 9-13; Hyderabad, India. Poster 0042. (Pneumonia 2014, vol. 3, Special issue, p. 160).<br><br>Lobo PR, Azevedo J, Silva E, Escobar EC, Menezes APO, Salgado K, et al. Epidemiology of pneumococcal meningitis in the metropolitan region of Salvador, Brazil, before and after the introduction of PCV10. Poster session: Controlling Pneumococcal Disease Around the Globe at: 9th International Symposium on Pneumococci and Pneumococcal Diseases; 2014 Mar 9-13; Hyderabad, India. Poster 0023. (Pneumonia 2014, vol. 3, Special issue, p. 161).<br><br>Lopez EL, Glatstein E, Ezcurra GC, Teplitz E, Iacono M, Garnero A, et al. Impact of universal pneumococal vaccination in children in Argentina. Poster Session: Vaccines: Pneumococcal at: IDWeek 2014; 2014 Oct 8-12; Philadelphia, PA, USA; 2014 [cited 2016 Jul 8]. Poster 1096, p. 324. Available from: https://idsa.confex.com/idsa/2014/webprogram/Paper46687.html<br><br>Machado K, Algorta G, Varela A, Amaya G, Assandri E, Mota MI, et al. Serotype 3 streptococcus pneumoniae invasive pneumococcal diseases and pneumonia in a referral children hospital, 1998 to 2014. Poster session: Severe Childhood Bacterial Infections at: 9th World Conference of the World Society for Pediatric Infectious Diseases; 2015 Nov 18-21; Rio de Janeiro, Brazil; 2015 [cited 2016 Jul 7]. Abst 0453, p. 374. Available from: http://wspid.kenes.com/Documents/WSPID%20All%20Abstracts.pdf<br><br>Silva E, Santos MS, Escobar EC, Azevedo J, Galvão VS, Silvany A, et al. Molecular epidemiology of non-vaccine pneumococcal serotypes isolated from meningitis patients in Salvador, Brasil, prior and after the introduction of PCV10. Poster Session at: 8th International Symposium on Pneumococci and Pneumococcal Disease; 2012 March 11–15; Iguaçu Falls, Brazil; 2015 [cited 2016 Jul 7]. Poster 264, p. 295. Available from: http://www2.kenes.com/ISPPD/Scientific/Documents/FinalAbstractbook.pdf	

Berezin EN, Safadi MA, de Paula A, Arantes A, Sini R, Guerra ML, et al. Hospital-based surveillance of serotypes associated with pneumococcal invasive disease among children < 5 years in Sao Paulo and Uberlandia Brazil. Paper presented at: 7th World Conference of the World Society for Pediatric Infectious Diseases; 2011 Nov 16-19; Melbourne, Australia. Abst 779.<br><br>Hortal M. , Estevan M, Laurani H, Iraola M, Meny M. Decline in pediatric pneumonia hospitalizations following PCV7 and PCV13 introduction in Uruguay. Poster Session at: 8th International Symposium on Pneumococci and Pneumococcal Disease; 2012 March 11–15; Iguaçu Falls, Brazil; 2012 [cited 2016 Jul 7]. Poster 245, p. 276. Available from: http://www2.kenes.com/ISPPD/Scientific/Documents/FinalAbstractbook.pdf<br><br>Liphaus BL, Okai MIG, Yu ALF, Ribeiro AF, Carvalhanas TRMP. Impact of 10-valent pneumococcal conjugate vaccine routine immunization schedule in incidence of pneumococcal meningitis in the state of São Paulo. Poster Session at: 8th International Symposium on Pneumococci and Pneumococcal Disease; 2012 March 11–15; Iguaçu Falls, Brazil; 2012 [cited 2016 Jul 7]. Poster 11, p. 322. Available from: http://www2.kenes.com/ISPPD/Scientific/Documents/FinalAbstractbook.pdf<br><br>Pírez MC, Algorta G, Cedrés A, Sobrero H, Varela A, Giachetto G, et al. Impact of universal pneumococcal vaccination (PCV7v/PCV13v) on hospitalizations for pneumonia and meningitis in children Hospital Pediatrico-Centro hospitalario Pereira Rossell, Uruguay. Poster Session at: 8th International Symposium on Pneumococci and Pneumococcal Disease; 2012 March 11–15; Iguaçu Falls, Brazil; 2012 [cited 2016 Jul 7]. Poster 258, p. 289. Available from: http://www2.kenes.com/ISPPD/Scientific/Documents/FinalAbstractbook.pdf	Short post vaccination period
Algorta G, Cedres A, Sobrero H, Varela A, Giachetto G, Pirez MC. Pneumococcal meningitis before and after pneumococcal conjugate vaccine 7 and 13 valent universal vaccination. 2005-2012. CHPR. Montevideo Uruguay. Paper presented at: XV Congresso Latino-americano de Infectologia Pediátrica – SLIPE 2013; XV Jornada Nacional de Imunização da SBIm; 2013 June 26-29; São Paulo, Brazil; 2013 [cited 2016 Jul 8]. Abst OR-036, p. 36. Available from: http://www.slipe.org/congreso/resumen22013.pdf.	No differentiation between vaccines

Vargas Sandoval G, Catañeda-Orjuelas CA, Castañeda E, Agudelo CI, De la Hoz-Restrepo F. Pneumococcal conjugate vaccine impact on children mortality: an analysis of data from Colombia, 2002-2012. Paper presented at: 9th World Conference of the World Society for Pediatric Infectious Diseases; 2015 Nov 18-21; Rio de Janeiro, Brazil; 2015 [cited 2016 Jul 7]. Abst 0779, p. 59. Available from: http://wspid.kenes.com/Documents/WSPID%20All%20Abstracts.pdf	
Jarovsky D, Brandileone MCC, Almeida SCG, Almeida RJS, Morais JC, Almeida FJ, et al. PCV10 impact on streptococcus pneumoniae serotypes distribution: a seven-year hospital-based surveillance study in invasive pneumococcal disease. Paper presented at: 33rd Annual European Society for Paediatric Infectious Diseases Meeting; 2015 May 12-16. Leipzig, Germany; 2015 [cited 2016 Jul 7]. Abst. 0879, p. 851. Available from: http://espid2015.kenes.com/Documents/ESPID%202015%20Abstracts.pdf	Denominator not based on population
Safadi MA, Berezin EN, Almeida F, Arnoni M, Guerra ML, Brandileone MC. Hospital-based surveillance to evaluate the early effects of pneumococcal vaccination program in Sao Paulo. Poster Session: Controlling Pneumococcal Disease Around the Globe at: 9th International Symposium on Pneumococci and Pneumococcal Diseases; 2014 Mar 9-13; Hyderabad, India. Poster 0552. (Pneumonia 2014, vol. 3, Special issue, p. 196).	No denominator
Pirez MC, Algorta G, Chamorro F, Romero C, Varela A, Cedres A, et al. Changes in hospitalizations for pneumonia after universal vaccination with pneumococcal conjugate vaccines 7/13 valent and haemophilus influenzae type b conjugate vaccine in a Pediatric Referral Hospital in Uruguay. Pediatr Infect Dis J. 2014;33(7):753-9. doi: 10.1097/inf.0000000000000294. PubMed PMID: 24492286.<br><br>Toscano C, Afonso ET, Nunes S, Minamisava R, Cristo EB, Morais-Neto OL, et al. Impact of 10-valent pneumococcal conjugate vaccine on the economic burden of pneumonia hospitalizations in children in Brazil. Poster Session at: 9th World Conference of the World Society for Pediatric Infectious Diseases; 2015 Nov 18-21; Rio de Janeiro, Brazil; 2015 [cited 2016 Jul 7]. Abst 0884, p. 118. Available from: http://wspid.kenes.com/Documents/WSPID%20All%20Abstracts.pdf	Did not present data on effectiveness
dos Santos SR, Passadore LF, Takagi EH, Fujii CM, Yoshioka CR, Gilio AE, et al. Serotype distribution of Streptococcus pneumoniae isolated from patients with invasive pneumococcal disease in Brazil before and after ten-pneumococcal conjugate vaccine implementation. Vaccine. 2013;31(51):6150-4. doi: 10.1016/j.vaccine.2013.05.042. PubMed PMID: 23747454.	Pre and post comparison based on proportions

Fernández V JP, Goecke H C, von Borries C, Tapia R N, Santolaya de P ME. Incidencia de egresos por neumonía en niños menores de 24 meses antes y después de la implementación de la vacuna conjugada antineumocócica 10-valente en el Programa Nacional de Inmunizaciones de Chile. Rev Chil Pediatr. 2015;86(3):168-72. doi: 10.1016/j.rchipe.2015.04.026. PubMed PMID: 26363857.	
Pirez M, Giachetto G, Algorta G, Cedres A, Sobrero H, Sanchez Varela M, et al., editors. Pneumonia and meningitis hospitalizations before and after pcv implementation in the national vaccination schedule in uruguay. Poster Session: Controlling Pneumococcal Disease Around the Globe at: 9th International Symposium on Pneumococci and Pneumococcal Diseases; 2014 Mar 9-13; Hyderabad, India. Poster 0247. (Pneumonia 2014, vol. 3, Special issue, p. 152).<br><br>Pirez MC, Algorta G, Chamorro F, Cedres A, Sobrero H, Varela A, et al. Community adquired pneumonia etiology before and after universal vaccination with pneumococcal conjugate vaccines and H.ifluenzae b conjugate vaccine. Hospital Pediatrico-Centro Hospitalario Pereira Rossell Uruguay 2003-2011. Paper presented at: XV Congresso Latino-americano de Infectologia Pediátrica – SLIPE 2013; XV Jornada Nacional de Imunização da SBIm; 2013 June 26-29; São Paulo, Brazil; 2013 [cited 2016 Jul 8]. Abst OR-030, p. 30. Available from: http://www.slipe.org/congreso/resumen22013.pdf	Hospital discharge as denominator
Gentile A, del Valle Juarez M, Lucion MF, Martinez AC, Romanin V, Giglio N, et al. Influence of respiratory viruses on the evaluation of 13-valent pneumococcal conjugate vaccine effectiveness in children under 5 years old. A time-series study 2001-2014. Poster session Vaccines: PCV at: IDWeek 2015; 2015 Oct 7-11; San Diego, California; 2015 [cited 2016 Jul 8]. Poster 1765, p. 1888. Available from: https://idsa.confex.com/idsa/2015/webprogram/Paper51879.html	Incomplete data
Gentile A, Juarez MV, Lucion MF, Romanin VS, Giglio N, Bakin J. Influencia de los virus respiratorios en la evaluación de la efectividad de la vacuna neumocócica de 13 serotipos en menores de 5 años: estudio de series temporales 2001-2013. Arch Argent Pediatr. 2015;113(4):310-6. doi: 10.1590/s0325-00752015000400006. PubMed PMID: 26172005.	Absolute Number of cases only
