# Supplementary material for: Impact and Effectiveness of 10 and 13-Valent Pneumococcal Conjugate Vaccines on Hospitalization and Mortality in Children Aged Less than 5 Years in Latin American Countries: A Systematic Review
Source: PLoS One. 2016 Dec 12;11(12):e0166736. doi: 10.1371/journal.pone.0166736 (PMC5152835; doi:10.1371/journal.pone.0166736)
Supplement: S1 Table — (DOCX) [file pone.0166736.s006.docx]

**S1 Table**: Risk of bias assessment of pneumonias endpoint.

| First author, year | Country | Vaccine/ schedule | Study design | Methodological Quality - Comments |
| --- | --- | --- | --- | --- |
| Afonso, 2013 [25] | Brazil | PCV-10 | Interrupted time series | i) The study is performed in five out of 10 capital cities of Brazil originally planned, and therefore representativeness of results to the country as a whole is limited; ii) Data from only one year post vaccine introduction is considered in the analysis; iii) Outcome definition with low specificity; potential misclassification of ICD; iv) Limitations inherent to ecological studies. |
| Suarez, 2016 [24] | Peru | PCV-10 | Interrupted time series | i) Results are reported for outpatient and hospitalized pneumonia combined, as it is reported into the data source considered; ii) Inconsistency of results based on RENACE data and national hospitalization database, attributed to poor data quality, limited representativeness, and coverage over time; iii) Limitations inherent to ecological studies. |
| Becker-Dreps, 2014 [21] | Nicaragua | PCV-13 | Interrupted time series | Transition period not considered; short post-vaccination period; outpatient and inpatient data pooled together. |
| Diaz, 2016 [22] | Chile | PCV-10 | Nested case-control | i) The endpoint considered (J13-18), although more specific as viral J12 codes are excluded, limited the comparability with other studies, most of which consider an outcome with more sensitive case definition (J12-J18); ii) Missing mortality data particularly in vaccinated children (VE was higher against all cause mortality than against pneumonia hospitalizations and deaths); iii) Misclassification of vaccination status in the 2010 cohort. |
| Hortal, 2015 [18] | Uruguay | PCV-13 | Cohort | i) The study is performed in one Region in Uruguay, and therefore data representativeness is questionable. ii) We assumed that the hospitals in which cases were captured covers the whole area and thus population based incidence estimates are available. However, this is not clearly reported in the paper. iii) The methods section of the study is very succinct not allowing for the understanding of several methodological aspects of the analysis. iv) It is not clear how denominator data was estimated. v) Losses to follow-up and control of confounding were not mentioned. |
| Sgambatti, 2016 [28] | Brazil | PCV-10 | Before-after | i) The study is performed in one municipality in Brasil and therefore data representativeness is questionable; ii) Increase in pediatric hospital beds during the study period; iii Limitations inherent to ecological before-after studies. |
| Scotta, 2014 [27] | Brazil | PCV-10 | Before-after | i) The authors did not present p-values or confidence limits of effectiveness estimates; ii) Inherent to before-after studies, existing trends prior to the intervention and seasonality patterns are not considered when comparing rates before and after vaccine introduction. |
| Hortal, 2014 [17] | Uruguay | PCV-13 | Before-after | i) The study is performed in one Region in Uruguay, and therefore data representativeness is questionable; ii) Data for the pre-vaccine introduction period is for 2001-2004, that is, long before vaccine was introduced (March 2008), and is obtained from a previous study (which is not cited). Further, this data is presented in an aggregate manner; iii) The authors report that hospital based surveillance resulting in the data used for the post-vaccine introduction period was initiated in 2009 - it is thus questionable whether the same methods and catchment population were used in the pre and post vaccine periods; iv) We assumed that the hospitals in which cases were captured covers the whole area and thus population based incidence estimates are available. However, this is not clearly reported in the paper; v) It is not clear how denominator data was estimated - considering results presented it appears that the authors considered a single population estimate for 2011 for estimating incidence rates in the pre and post intervention periods; vi) A study published by the same authors in the same study location, using a cohort methodology, resulted in effectiveness results 50% higher than the estimates reported in this study. vii) Inherent to before-after studies, existing trends prior to the intervention and seasonality patterns are not considered when comparing rates before and after vaccine introduction. viii) Confidence intervals for the estimates were not presented. |
| Gentile, 2015 [30] | Argentina | PCV-13 | Before-after | i) The study is performed in one municipality in Argentina, and therefore data representativeness is questionable; ii) Results are reported for 23% of outpatient and hospitalized pneumonia combined, from four reference hospitals; iii) Data for the pre-vaccine introduction period is for 2003-2005, that is, long before vaccine was introduced Jan 2012). Further, this data is presented in an aggregate manner; iii) It is not clear how population denominator data were generated for both the pre and post vaccine periods - considering results presented it appears that the authors considered a single population estimate for 2000 for estimating incidence rates in the pre intervention period, and a single estimate for 2010 for estimating rates for the post intervention period (2012 and 2013); iv) Inherent to before-after studies, existing trends prior to the intervention and seasonality patterns are not considered when comparing rates before and after vaccine introduction. |
| Gaiano, 2013 [29] | Argentina | PCV-13 | Before-after | i) We assumed that the results are reported for outpatient and hospitalized pneumonia combined, but this is not clearly indicated by the authors; ii) It is not clear how pneumonia cases are captured; iii) Data from only one year (2011) is considered in the pre-intervention period; iii) Data from the vaccine introduction year (2012) was further excluded from the analysis in the effectiveness estimates calculated by the reviewers. Data for 2013 considered for the pre vaccine introduction period includes only data for Jan-July; viii) Vaccine coverage was not disclosed. |
| Rearte, 2015 [31] | Argentina | PCV-13 | Before-after | i) The study is performed in one municipality in Argentina (Concordia), and therefore data representativeness is questionable; ii) Not clear if cases considered in the study included both outpatient and hospitalized pneumonia cases or only hospitalized; iii) Authors indicate that surveillance was performed in 4 hospitals in which X-ray equipment were available. It is thus questionable whether these hospitals in which cases were captured cover the whole area and thus population based incidence estimates are available; iv) Data for the pre-vaccine introduction period are for 2002-2005, that is, long before vaccine was introduced Jan 2012). It is not clear whether the same hospitals and same methods were used in the study in which data for the pre-intervention period were collected. Further, these data are presented in an aggregate manner; v) Data for only one year (April 2014 - March 2015) in the post intervention period are considered in the analysis; vi) It is not clear how population denominator data were generated for both the pre- and post-vaccine periods; vii) Inherent to before-after studies, existing trends prior to the intervention and seasonality patterns are not considered when comparing rates before and after vaccine introduction; viii) Vaccine coverage was not disclosed. |
