# Supplementary material for: Impact and Effectiveness of 10 and 13-Valent Pneumococcal Conjugate Vaccines on Hospitalization and Mortality in Children Aged Less than 5 Years in Latin American Countries: A Systematic Review
Source: PLoS One. 2016 Dec 12;11(12):e0166736. doi: 10.1371/journal.pone.0166736 (PMC5152835; doi:10.1371/journal.pone.0166736)
Supplement: S2 Table — (DOCX) [file pone.0166736.s007.docx]

**S2 Table**: Risk of bias assessment of invasive pneumococcal disease endpoint.

| First author, Year | Country | Vaccine | Study Design | Risk of bias |
| --- | --- | --- | --- | --- |
| Valenzuela, 2014 [45] | Chile | PCV-10/3+1;2+1 | Before-After | i) Denominator population used for rate estimation not shown by authors and from methods it is not possible to conclude whether annual population estimates were used. ii) Although the authors report conducting a time series analysis, in methods it is indicated that p-value for trend was used. In results, % reduction in rates and respective confidence limits are reported. Thus, it does not appear that analysis took into account existing trends and seasonality patterns prior to the intervention, and therefore the analysis performed characterizes this as a before-after study. iii) vaccination coverage was not disclosed. iv) based on secondary data. |
| Andrade, 2016 [32] | Brazil | PCV-10/3+1; catch-up | Interrupted time series | i) Overall and serotype specific vaccine impact is reported. Serotype data only available for approximately 5% of cases reported to one of the two different surveillance databases (laboratory database) which were linked and considered in this study. ii) short pre-vaccination period. iii) based on passive surveillance, which was increased following vaccination. iv) over-representation of São Paulo state in data from Instituto Adolfo Lutz (IAL). |
| Domingues, 2014 [15] | Brazil | PCV-10/3+1; catch-up | Case control | i) Study was conducted in 10 of the 27 states of Brazil, which accounted for 66% of the National population. ii) variable laboratory capacity across states; iii) differential response rates for cases and controls. iv) overrepresentation of meningitis. |
| Garcia Gabarrot, 2014 [44] | Uruguay | PCV-13/ 2+1 | Before-after | i) Although the authors report this as a retrospective population based cohort study, the reviewers characterized this as a before-after study as rates for the pre- and post-intervention periods are compared and study population is not individually followed prospectively as would characterize a cohort study design. ii) Data considered in this analysis were obtained from the National Public Health Reference Laboratory for S. pneumoniae surveillance – it is not clear whether data included all IPD cases in the country, as the system is a passive laboratory based non-mandatory system. iii) Denominator population used for rate estimation not shown by authors, and from methods it is not possible to conclude whether annual population estimates were used. iv) Data for the pre- and post-vaccine introduction periods are presented in an aggregate manner. v) Inherent to before-after studies, existing trends prior to the intervention and seasonality patterns are not considered when comparing rates before and after vaccine introduction. vi) Case detection enhanced in post-vaccination period. |

Hanquet G, Valenciano M, Simondon F &Moren A. Vaccine effects and impact of vaccination programmes in post-licensure studies. Vaccine 2013; 31:5634– 5642.

Reeves BC, Deeks JJ, Higgins JPT, Wells GA. Chapter 13: Including non-randomized studies. In: Higgins JPT, Green S (editors), Cochrane Handbook for Systematic Reviews of Interventios. Chichester (UK): Johns Wiley & Sons, 2008.

Sanderson S, Tatt ID and Higgins JPT. Tools for assessing quality and susceptibility to bias in observational studies in epidemiology: a systematic review and annotated bibliography. International Journal of Epidemiology 2007; 36:666-676.

Sterne JAC, Egger M, Moher D (Editors). Chapter 10: Addressing reporting bias. In: Higgins JPT, Green S (editors), Cochrane Handbook for Systematic Reviews of Interventions. Chichester (UK): Johns Wiley & Sons, 2008.

Wells GS, O’Connell D, Peterson J, Welch V, Losos M, Tugwell P: The Newcastle-Ottawa scale (NOS) for assessing the quality of nonrandomized studies in meta-analyses. Ottawa Hospital Research Institute. 2008

[http://www.ohri.ca/programs/clinical_epidemiology/oxford.asp]
